# Transcriptomic analysis of interstock-induced dwarfism in Sweet Persimmon (*Diospyros kaki* Thunb.)

**DOI:** 10.1038/s41438-019-0133-7

**Published:** 2019-05-01

**Authors:** Yanying Shen, Weibing Zhuang, Xutong Tu, Zhihong Gao, Aisheng Xiong, Xinyi Yu, Xuehan Li, Feihong Li, Shenchun Qu

**Affiliations:** 1 College of Horticulture, Nanjing Agricultural University, 210095 Nanjing, Jiangsu China; 2 Jiangsu Key Laboratory for Horticultural Crop Genetic Improvement, 210014 Nanjing, China; 3 Jiangsu Key Laboratory for the Research and Utilization of Plant Resources, Institute of Botany, Jiangsu Province and Chinese Academy of Sciences, 210014 Nanjing, China

**Keywords:** Plant hormones, Plant development, RNA sequencing

## Abstract

Growth monitoring indicated that the height of ‘Kanshu’ plants with ‘Nantong-xiaofangshi’ as an interstock was significantly shorter than that of ‘Kanshu’ plants with no interstock. A transcriptome analysis of the two graft combinations (‘Kanshu’/*Diospyros lotus* and ‘Kanshu’/‘Nantong-xiaofangshi’/*Diospyros lotus*) was conducted to explore the dwarfing genes related to the use of the ‘Nantong-xiaofangshi’ interstock. Hormone levels and water conductance were also measured in these two graft combinations. The results indicated that the levels of both IAA and GA were lower in ‘Kanshu’ that had been grafted onto the ‘Nantong-xiaofangshi’ interstock than in ‘Kanshu’ with no interstock; additionally, the water conductance was lower in grafts with interstocks than in grafts without interstocks. The expression of *AUX/IAA* and auxin-responsive *GH3* genes was enhanced in scions grafted on the interstock and was negatively correlated with the IAA content and growth of scions. The expression of *GA2ox*, *DELLA*, and *SPINDLY* genes were also upregulated and associated with a decrease in the level of GA in scions grafted on the interstock. Since one of the *GA2ox* unigenes was annotated as *DkGA2ox1* in *Diospyros kaki*, but was not functionally validated, a functional analysis was conducted in transgenic tobacco. Overexpression of *DkGA2ox1* in transgenic plants resulted in a dwarf phenotype that could be recovered by the exogenous application of GA_3_. We conclude that the ‘Nantong-xiaofangshi’ interstock affects the water conductance and expression of genes related to the metabolism and transduction of IAA and GA in the grafted scion and thus regulates phytohormone levels, producing dwarfing.

## Persimmon: Interstock alters water flow and hormone metabolism, prompting dwarfism

The use of an interstock from a dwarf cultivar lowers the water conductance and affects hormone signal metabolism and transport of the sweet persimmon (*Diospyros kaki* Thunb.), resulting in smaller and more manageable fruit-bearing trees. Shenchun Qu from Nanjing Agricultural University, China, and colleagues compared ‘Kanshu’ persimmon scions grafted on a ‘*Diospyros lotus*’ rootstock with and without an interstock from a rare dwarf cultivar called ‘Nantong-xiaofangshi’. The interstock-grafted plants moved water less efficiently through their stems. The same plants also exhibited gene activity patterns that correlated with lower levels of the key growth hormones auxin and gibberellic acid. Expressing one of those putative hormone-repressor genes in transgenic tobacco caused the plants to grow shorter. By helping elucidate the mechanisms of interstock-induced dwarfing, the findings could help improve future persimmon cultivation.

## Introduction

Oriental or sweet persimmon (*Diospyros kaki* Thunb., 2n = 6X= 90), also called Japanese persimmon or kaki, is believed to have originated in China. Because sweet persimmon is one of the tallest fruit-tree species, the management of its trees is very labor-intensive. Therefore, the identification and use of a dwarfing rootstock or interstock could be used to control tree vigor and reduce the amount of labor required^1^ for management. In fact, the use of dwarfing rootstocks or interstocks is the primary approach for producing dwarfed fruit trees because these rootstocks and interstocks result in reduced tree volume, height, canopy diameter, and circumference^2^. Some persimmon cultivars that have potential dwarfing effects on grafted scions could be used as rootstocks or interstocks^1,3^. The dwarfed persimmons obtained by the use of dwarfing rootstocks or interstocks can be used to establish orchard systems that are easier to manage than systems without dwarfed persimmons, thus reducing labor demands while maintaining a high yield efficiency^4^.

Studies on the mechanism of scion dwarfing have been conducted in fruit trees, such as apple^5^, pear^6^, peach^7^, and citrus^8^. Current hypotheses of the dwarfing mechanisms of rootstocks and interstocks that alter scion size suggest that hydraulic properties^9^, nutrients^10^, phenols^11^, enzymatic activity^12^, and phytohormones^13^ all may play a role. Some studies have indicated that the influence of these factors is dependent on the nature of the graft union anatomy^14^ and resistivity^15^. However, some studies have reported that the degree of graft union compatibility is not affected the translocation and transport of nutrients and ions^16,17^. It is well known that hormones play an important role in regulating tree size. The dwarfing effect of specific rootstocks or interstocks is due to alterations in the expression of genes associated with hormone metabolism and transduction, which in turn may regulate the balance of endogenous hormones in scions^18,19^.

Rootstocks can influence the metabolism and transport of IAA in grafted scions. Early studies found that the basipolar transport of IAA in dwarfing rootstocks was lower than that in semidwarfing rootstocks^20^. Lochard and Schneider^15^ postulated that a dwarfing rootstock could affect the basipetal transport of IAA in the phloem and cambium of the rootstock stem, resulting in a decreased supply of IAA to roots^11^. GA is also related to plant growth and development and plays a direct role in stem elongation growth^21^. Previous research provided evidence that GAs can exhibit nonpolar transport over long distances in grafted plants and affect growth^22^. Hooijdonk et al.^23^ suggested that the transmission of GAs is suppressed by the reduction in the basipetal transport of IAA to the root and that this might decrease the duration of elongation growth. Moreover, root-produced GA_19_ supplied to the scion, a potential precursor of bioactive GA_1_, which is required for shoot extension, is restricted by dwarfing rootstocks^24^. Interstocks can also effectively govern the growth of grafted plants^25^. A study examining the dwarfing mechanism of interstocks indicated that interstocks can affect the levels of some hormones and gene expression in the stem^26^. Persimmons grafted onto a dwarfing interstock can effectively control scion growth^27^; however the mechanism responsible for the dwarfing effect of interstocks is still not clearly understood.

Many studies have demonstrated that grafting can alter gene expression in scions. Jensen et al.^5^ reported the gene expression patterns in ‘Gala’ apple scions grafted onto different rootstocks and identified genes potentially associated with horticulturally important traits. Prassinos et al.^28^ found that the timing of differentially expressed genes (DEGs) coincided with the early cessation of terminal shoot growth in a scion/rootstock combination and suggested that these DEGs might be involved in the dwarfing phenomenon. Vascular tissues theoretically provide opportunities for the long-distance translocation of micromolecules and macromolecules^29^. The presence of mobile mRNA in the phloem was first reported in potato^30^. Subsequently, a series of transported mRNA studies in phloem tissues were conducted in a variety of species, including Arabidopsis^31^, pumpkin^32^, pear^33^, and apple^34^. Other studies have demonstrated through grafting experiments that certain mRNAs can be transported in the vascular system based on a noncellular autonomous pathway mechanism^35^. Additional studies documented the translocation of endogenous mRNAs, including *GAI*^36,37^, *AUX/IAA14*^34^, *KNOTTED1*^38^, *NAC*^39^, and *StBEL5*^40^, where *GAI*, *AUX/IAA14*, and *NAC* mRNAs exhibited bidirectional transport in grafted plants. Collectively, these studies have demonstrated that mRNAs are not only transported across cellular interfaces but also function as long-distance signaling molecules and thus may play a significant role in the regulation of plant physiology and development^34,41^. Grafting experiments have also confirmed that the rootstock-scion transport of mutated gene mRNA induces phenotypic changes^42,43^.

As recorded in 1982 in the ‘Fruit Tree Resources Survey of Jiangsu Province’, Nantong City (Rudong district and Hai’an district) China, ‘Nantong-xiaofangshi’ persimmon (*Diospyros kaki* Linn. cv. Nantong-xiaofangshi) exhibits many advantageous characteristics. These characteristics include significant dwarfing, robust growth, high adaptability, early bearing, high and stable yield, excellent fruit quality, and astringency that is relatively easy to remove^44^. Although the dwarfing ability of ‘Nantong-xiaofangshi’ persimmon is much greater than that of the two persimmon cultivars ‘Dafangshi’ and ‘Zhushahong'^45^, the seeds of ‘Nantong-xiaofangshi’ are rare, and it is difficult for the cuttings and tissue culture seedlings of ‘Nantong-xiaofangshi’ to root. However, due to its excellent horticultural traits, ‘Nantong-xiaofangshi’ has been used as a dwarfing interstock in persimmon, even though the mechanisms responsible for the dwarfing effect of interstocks have not yet been elucidated.

In the present study, we examined the entire vascular system of persimmon stems relative to the impact of a dwarfing interstock. RNA-seq analysis of ‘Kanshu’/*Diospyros lotus* and ‘Kanshu’/‘Nantong-xiaofangshi’/*Diospyros lotus* was conducted to select candidate unigenes associated with the dwarfing effect induced by using ‘Nantong-xiaofangshi’ as an interstock. The results indicated that some unigenes were concentrated in hormone signal metabolism and transduction. Therefore, the levels of endogenous IAA, GA, and ABA were measured in the same tissues. In GA metabolism, *GA2ox* can catalyze bioactive GA and convert it to an inactive product. The functional role of *GA2ox* genes in dwarfing has been previously demonstrated in several species^46,47^. Since a unigene in *Diospyros kaki* was annotated as *DkGA2ox1* but has not been functionally confirmed, we examined the effect of *DkGA2ox1* in transgenic tobacco. We also investigated the transport of endogenous *DkGA2ox1* transcripts from ‘Nantong-xiaofangshi’ interstock tissues to the scion. Our study provides molecular information that helps clarify the dwarfing mechanism associated with the use of ‘Nantong-xiaofangshi’ as an interstock in persimmon.

## Materials and methods

### Plant material

Taking into consideration current production practices, this study utilized nongrafted and grafted combinations of persimmon, including *Diospyros lotus*, ‘Kanshu’, ‘Nantong-xiaofangshi’/*Diospyros lotus*, ‘Kanshu’/*Diospyros lotus*, and ‘Kanshu’/‘Nantong-xiaofangshi’/*Diospyros lotus* (‘Nantong-xiaofangshi’, hereafter referred to as N.x). Seedlings with uniform growth were selected for grafting. The first grafting (rootstock grafted with interstock) was conducted in early April 2014 with 2-year-old *Diospyros lotus* and 1-year-old N.x used as the rootstock and interstock, respectively. The second branch grafting (interstock grafted with scion) was conducted in May 2015 with 1-year-old ‘Kanshu’ branches used as scions. At the same time, combinations of ‘Kanshu’/*Diospyros lotus* and N.x/*Diospyros lotus* were made. The grafted ‘Kanshu’ persimmon plants were cultivated in soil in pots and subjected to conventional management outdoors. The experimental materials were obtained from the Introduction and Breeding Base in Kunshan (Jiangsu, China).

All persimmon stem tissues were sampled at the end of April. The tissue samples (combined phloem and xylem) were used in various analyses, and each experimental group (graft combination) contained three biological replicates that were sampled during the active growth period of persimmon. The internal tissues of the stem samples were exposed by slicing the stems with a scalpel. The collected samples were placed in 5 ml tubes and immediately frozen in liquid nitrogen and stored at −80 °C for further use.

### Transcriptome sequencing (RNA-seq)

**Fig. 1:**
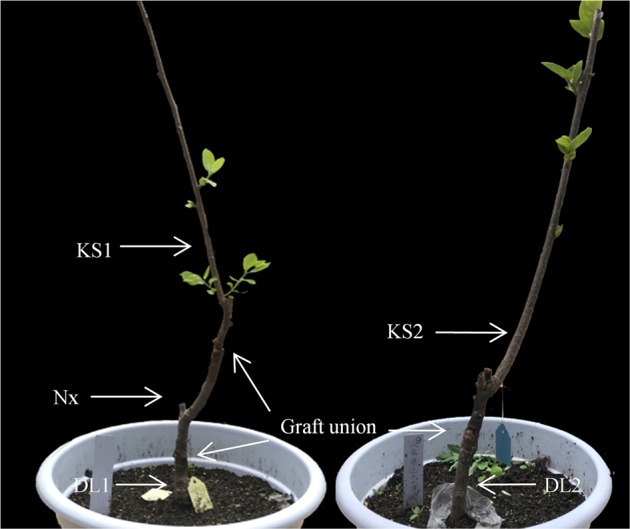
Stem tissues of the two graft combinations prepared for transcriptomic sequencing and analyses. KS1, Nx, and DL1: scion, interstock and rootstock in the combination of ‘Kanshu’/N.x/*Diospyros lotus*; KS2 and DL2: scion and rootstock in ‘Kanshu’/*Diospyros lotus*

Transcriptomic sequencing and analyses were conducted on stem tissues obtained from the scion (10 cm above the graft union between the interstock and scion), interstock (middle of the interstock), and rootstock (5 cm from the graft union) of two graft combinations, i.e., from the combination of ‘Kanshu’/N.x/*Diospyros lotus* and the scion and rootstock from ‘Kanshu’/*Diospyros lotus*, during the rapid elongation period (at the end of April) (Fig. 1). Before being transcriptome was sequenced, the cDNA libraries obtained from three individuals of each graft combination were separately mixed. Sequencing was performed using an Illumina HiSeq™ 4000 platform in paired-end mode with a read length of 150 bp by 1GENE Science and Technology Co., Ltd. (1GENE Tech, Hangzhou, China). A total of 7.7–8.4 Gb of raw bases were obtained from each library. After quality assessment of the obtained sequence data, adaptor sequences were removed, and raw reads with more than 20% low-quality bases (quality < 20) and reads containing >5% Ns were discarded. The remaining reads were considered clean reads. Trinity software (http://trinityrnaseq.sourceforge.net) was utilized for the de novo assembly of the transcriptome short reads^48^. The assembly parameters were set to ‘min_contig_length = 200, min_kmer_cov = 2, min_glue = 3, and seqType = fq’; all other parameters set to default values. Ultimately, the contigs were clustered into unigenes with tgicl^49^.

The unigenes from *D. kaki* Thunb. in these two graft combinations were analyzed to select unigenes associated with dwarfing. Since the reference genome of persimmon has not yet been published, the sequences were annotated and classified by BLASTx (Version: BLAST-2.2.28) analysis with a threshold of *E*-value < 10^−5^ according to the NCBI nonredundant (Nr) database (as of March, 2016), SwissProt, Clusters of Orthologous Groups (COG) and Kyoto Encyclopedia of Genes and Genomes (KEGG)^50^. Based on the Nr database annotations, Blast2GO^51^ and WEGO^52^ were used for Gene Ontology (GO) annotation and functional classification with the default parameters. Fragments per kb per million fragments (FPKM) was used to avoid the bias caused by different gene lengths^53^.

### Phytohormone levels

Phytohormones were extracted from stems of the two grafted combinations at the same sampling positions as those described in ‘Transcriptome Sequencing (RNA-seq)’. Three biological replicates were used for the determination of each hormone. The levels of gibberellin (GA_4_), indole-3-acetic acid (IAA), and abscisic acid (ABA) were determined by Zoonbio Biotechnology Co., Ltd. (Nanjing, China) using a modification of the protocol described by You et al.^54^. Approximately 0.5 g of fresh tissue was ground in liquid nitrogen, and 5 ml of extraction buffer composed of isopropanol/hydrochloric acid was added to each sample. The samples with extraction fluid were shaken at 4 °C for 30 min. Subsequently, 10 ml dichloromethane was added to each sample tube, and the samples were again shaken at 4 °C for 30 min. This was followed by centrifugation at 13,000 rpm for 5 min at 4 °C, after which the organic phase was extracted. The obtained organic phase was dried under N_2_ in the dark and then dissolved in 400 μl methanol (0.1% methanoic acid) and subsequently filtered through a 0.22 μm filter membrane. The purified product (2 μl of injection volume) was then subjected to high-performance liquid chromatography-tandem mass spectrometry (HPLC-MS/MS) analysis. HPLC analysis was performed using a poroshell SB-C18 (Agilent Technologies) column (2.1 mm × 150 mm, 2.7 μm). The mass spectrometry conditions were as follows: the spray voltage was 4500 V; the pressures of the air curtain, nebulizer, and aux gas were 15, 65, and 70 psi, respectively; and the atomizing temperature was 400 °C. The hormone contents were quantitated by the standard curve method and calculated as the average concentration × volume coefficient (add the volume of the solvent)/mass coefficient (the quality of the original sample).

### Hydraulic conductance

We selected graft plants with relatively uniform growth to measure water conductance using a high-pressure flow meter (HPFM-Gen3, Dynamax Inc., Houston, TX), with reference to the method described in Tyree et al.^55^. The stem was cut 5 cm below the graft union between the rootstock and interstock/scion. The stump was connected to the HPFM with a water-tight seal to measure water conduction resistance (R_canopy_). Next, the stem was cut 5 cm above the graft union between interstock and scion. The scion was connected to the HPFM to measure the resistance (R_scion_). For comparison, ‘Kanshu’/*Diospyros lotus* without interstock and ‘Kanshu’/‘Nantong-xiaofangshi’/*Diospyros lotus* were cut at the same position. The water conduction resistance of the grafting area was ‘R_canopy_–R_scion_'^56^. The water conductance and resistance were reciprocals of each other (K_h_ = 1/R).

### Reverse transcription-quantitative PCR (RT-qPCR)

Total RNA was extracted from samples of the scion stems of ‘Kanshu’ persimmon in the two different graft combinations (‘Kanshu’/N.x/*Diospyros lotus* and ‘Kanshu’/*Diospyros lotus*) using the Plant Total RNA Isolation Kit Plus (Foregene, Chengdu, China). The RNA quality and integrity were evaluated using a Nanodrop ND 1000 spectrophotometer (Nanodrop Technologies Inc., Delaware, USA) and agarose gel electrophoresis before the RNA was used for first-strand cDNA synthesis (TaKaRa, Dalian, China).

RT-qPCR was performed on an ABI Real-Time PCR System (Applied Biosystems, USA) using SYBR Premix Ex Taq™ (TaKaRa, Dalian, China). The 20 μl reaction system contained 10 μl SYBR Green I Mix, 1 μl diluted cDNA template (150 ng∙μl^−1^), 8.6 μl ddH_2_O, and 0.2 μl each of gene-specific upstream and downstream primers (10 μM). The following cycling conditions were used: an initial denaturation step of 95 °C (4 min), followed by 40 cycles of 95 °C (20 s), 60 °C (20 s), and 72 °C (40 s). Dissociation curves were generated from 60 °C to 95 °C to verify primer specificity with the presence of a single peak. Each reaction was conducted using three technical repeats and a negative control (water instead of cDNA template). All RT-qPCR reactions were carried out in accordance with the Minimum Information for Publication of Quantitative Real-Time PCR Experiments (MIQE) guidelines^57^. Primers for the persimmon tree were designed using Beacon Designer 7.0 (Premier Biosoft International, Palo Alto, CA, USA), and the specificity of the alignment was evaluated using BioEdit on the NCBI website, as well as our transcriptome database. Actin, *UBC*, *GADPH*, and *PP2A* genes served as reference genes for normalization (Table S1)^58^. Expression levels were calculated using the 2^−ΔΔCt^ method^59^. Each RT-qPCR evaluation of each gene utilized three biological replicates.

### Gene construct and tobacco transformation

The sense primer *DkGA2ox1*B-F containing a BamHI site and the antisense primer *DkGA2ox1*S-R containing a SalI site (Table 1) were utilized to create the *DkGA2ox1* mRNA plasmid construct. cDNA coding for *DkGA2ox1* was obtained from ‘Nantong-xiaofangshi’ using the following PCR conditions: initial denaturation at 94 °C for 4 min, followed by 35 cycles of 94 °C for 30 s, 62 °C for 40 s and 72 °C for 1 min 10 s, and then a final extension at 72 °C for 10 min. Amplified *DkGA2ox1* cDNA sequences were purified and subcloned into a PMD19-T simple vector (Takara, Dalian, China). The vector containing the *DkGA2ox1* cDNA was digested with BamHI and SalI and then integrated into the same sites of the transformation vector, pYH4215 (Fig. S1).

**Table 1:** Gene-specific primer pairs

Primers	Sequences (5′–3′)
*DkGA2ox1*B-F	5′-CGGGATCCATGGTGGTTTTATC-3'
*DkGA2ox1*S-R	5′- CGAGCTCTCACGAGGCGGCAAC-3'
*DkGA2ox1*-F1	5′-TCAAAGCCACTCTCCCACTGA-3'
*DkGA2ox1*-R1	5′-CTCGGGAAAGTGGGGAAAACC-3'
*DkGA2ox1*-S-F1	5′-TCTATCCCACCCGACGAAAG-3'
*DkGA2ox1*-S-R1	5′-AGTGGGGAAAACCATCACGAG-3'
*Nt GA20ox*-F	5′-TGCTTTCTTTCTTTGTCCAA-3'
*Nt GA20ox*- R	5′-CTCTGTAATGCTTCTGTGTAA-3'
*Nt GA3ox* -F	5′-AAGACTGATGTGGCTCAT-3'
*Nt GA3ox* -R	5′-TGTTAATATGGTAGAATCCGTATGT-3'
*Nt tubA1*-F	5′-CTTATGTTCCGTGGTGATG-3'
*Nt tubA1*-R	5′-TTGGTGGCTGATAGTTGA-3'

The recombinant plasmid was introduced into *Agrobacterium tumefaciens* (strain EHA105) using the freeze-thaw method^60^ and was subsequently used for tobacco transformation. Disks of plant tissues cut from tobacco leaves were inoculated with *Agrobacterium tumefaciens* (strain EHA105) carrying the plasmid pYH4215-*DkGA2ox1* containing genes for β-glucuronidase (GUS) and hygromycin resistance (HygR) on MS medium with BA 1.0 mg·l^−1^ and NAA 0.2 mg·l^−1^ (pH 5.8). After 2–3 days in the dark, explants were transferred to a selective MS medium containing 6-BA 1.0 mg·l^−1^, NAA 0.2 mg·l^−1^, Hyg 30 mg·l^−1^, and Cb 200 mg·l^−1^ (pH 5.8). Next, regenerated shoots 3–5 cm in height were cut and cultured in ½ MS medium containing IBA 0.2 mg·l^−1^, Hyg 30 mg·l^−1^, and Cb 200 mg·l^−1^ (pH 5.8) to promote root development. The initial selection of *DkGA2ox1* transgenic tobacco leaves was based on Hyg resistance and GUS staining.

### Transgenic tobacco phenotyping

The propagation of transgenic tobacco and nontransgenic tobacco lines occurred through vegetative reproduction. The plants were grown on agar medium and transplanted to soil in pots 30 days after germination. Eighty days after their placement in the greenhouse, three wild-type plants and three plants from each transgenic line were used to measure plant height, internodal length, stem diameter, and leaf length and width. Phenotypic measurements of the transgenic plants were undertaken using two independent lines (three plants from each line). The length:width ratio of the leaves was calculated. Mature, fully expanded functional leaves of the transgenic and wild-type tobacco were collected during the blooming period for the measurement of GA levels as previously described.

### Expression analysis of transgenic tobacco genes

Gene-specific primers were designed for tobacco *GA20ox* (Accession number AB109762) and *GA3ox* (Accession number AB032198) sequences registered at NCBI (Table 1). The primers were utilized to examine the expression of these genes in transgenic and nontransgenic tobacco plants. A *Tubulin* gene from tobacco was used as a reference gene for the normalization of expression data. The reaction system contained 10 μl SYBR Green I Mix, 1 μl diluted cDNA template (150 ng μl^−1^), 8.4 μl ddH_2_O, and 0.3 μl each of the forward and reverse primers (0.15 pmol∙l^−1^). The following cycling conditions were used: an initial denaturation step of 95 °C (4 min), followed by 40 cycles of 95 °C (20 s), 60 °C (20 s), and 72 °C (40 s). Each expression analysis utilized three biological and technical replicates. The expression analysis was conducted utilizing ABI Real-Time PCR System software and the 2^−ΔΔCt^ method.

### Cloning and sequencing of *DkGA2ox1* in different cultivars

The open reading frame (ORF) of *DkGA2ox1* in ‘Kanshu’, N.x, and *Diospyros lotus* was cloned. Gene-specific primers for *DkGA2ox1* were designed based on sequences from *Diospyros kaki* Linn. cv. Nantong-xiaofangshi deposited in the NCBI database (GenBank ID, KJ664180.1) and our own RNA-seq database.

PCR-amplified cDNA fragments from *Diospyros lotus*, ‘Nantong-xiaofangshi’ and ‘Kanshu’ using *DkGA2ox1*-F1 and *DkGA2ox1*-R1 primers were obtained and sequenced (Table 1). Amplified cDNA sequences were purified and subcloned into the pEASY^®^ - Blunt cloning vector (TransGen Biotech, Beijing, China) for sequencing.

### Cleaved amplified polymorphic sequence (CAPS) analysis

Restriction enzyme cleavage sites of *Diospyros lotus*, N.x, and ‘Kanshu’ were constructed, and a specific ApaI cut site was identified (Thermo Scientific, Waltham, MA, USA). RT-PCR products were amplified with the *DkGA2ox1*-S-F1 and *DkGA2ox1*-S-R1 primers (Table 1) according to the instructions of PrimerSTAR GXL Polymerase (Takara, Dalian, China) and using the following PCR conditions: initial denaturation at 98 °C for 5 min, followed by 30 cycles of 98 °C for 10 s, 60 °C for 15 s and 68 °C for 30 s, and a final extension at 68 °C for 10 min. The amplified products were digested with ApaI and separated by 16% (m/v) polyacrylamide gel electrophoresis (PAGE).

### Statistical analyses

Experimental data were subjected to analysis of variance (ANOVA) using SAS 8.0 software (SAS Institute, Inc., Cary, NC, USA). The phylogenetic analysis was performed in MEGA 6.0 software (MEGA, Tempe, AZ, USA)^61^. Additional data analyses were performed using Graphpad Prism 6.0 (GraphPad Software, La Jolla, CA, USA) and Microsoft Excel 2007 (Microsoft Corporation, Redmond, WA, USA).

## Results

### Effect of ‘Nantong-xiaofangshi’ on scion height

**Fig. 2:**
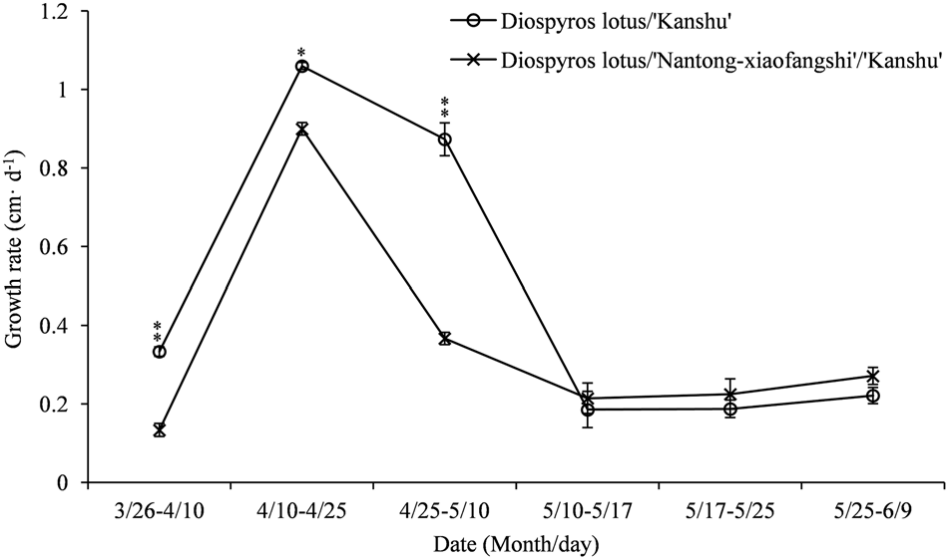
Changes in the ‘Kanshu’ scion growth rate for different graft combinations. Error bars indicate the standard error of five biological replicates. Asterisks indicate significant differences; one asterisk represents *P* < 0.05, and two asterisks represents *P* < 0.01

The initial scion length was 15 cm. After nearly one year, the scion length was measured from the top of the graft union to the shoot apex of the scion in each of the graft combinations beginning in late March 2016. The use of ‘Nantong-xiaofangshi’ as an interstock had a significant impact on the height of grafted trees (Fig. 2). After almost a year of growth, the shoot lengths of ‘Kanshu’ grafted on ‘Nantong-xiaofangshi’ interstocks were shorter than those grafted directly onto rootstocks. During the initial period of spring growth, the growth of ‘Kanshu’ scions without an interstock was significantly faster than the growth of plants grafted with an interstock. In the later stages of the growth period, after April 25, the growth of ‘Kanshu’ scions grafted on N.x. interstocks slowed dramatically relative to their growth on rootstocks without interstocks. There was no significant difference in growth rate between the two graft combinations after May 10.

### RNA-sequence analysis and functional annotation of persimmon transcripts in grafted trees

Gene expression in grafted persimmon trees was analyzed by RNA-seq in order to better understand the mechanisms underlying interstock-induced dwarfing. Five cDNA libraries were prepared from three tissues (scions, interstocks, and rootstocks) in the ‘Kanshu’/N.x/*Diospyros lotus* trees and from two tissues (scions and rootstocks) in the ‘Kanshu’/*Diospyros lotus* trees. The resulting cDNA libraries were sequenced on an Illumina HiSeq 4000 platform. After stringent quality evaluation and data filtering, 6.9–7.4 Gb of clean bases were obtained from each library (Table 2). The Q20, Q30 (sequencing error rate < 0.01), and GC content values were 98.32–98.48%, 95.90–96.25%, and 47.78–48.20%, respectively (Table 2). Contigs assembled from the high-quality reads were used to produce unigenes. Based on genetic divergence, ‘Kanshu’ and ‘Nantong-xiaofangshi’ belong to the same species (*Diospyros kaki*), and sequences from the tissues of the same species were assembled together. The sequence data were deposited in Figshare under 10.6084/m9.figshare.7406648SRP048768. This approach was used to maximize both the number and length of the unigenes that were obtained. Only data from *Diospyros kaki* were used to find candidate unigenes related to interstock-induced dwarfism. The length distribution of all transcripts is shown in Fig. S2. Ultimately, a total of 119,573 unigenes with a mean length of 662 nt were obtained.

**Table 2:** Summary of Illumina sequencing results

Samples	Total raw reads	Total clean reads	Total clean bases	Q20 percentage	Q30 percentage	GC percentage
KS2	56,025,676	49,604,844	7,440,726,600	98.32%	95.90%	47.78%
KS1	53,887,352	47,093,378	7,064,006,700	98.48%	96.25%	48.20%
IN	51,690,206	46 443 640	6,966,546,000	98.49%	96.27%	47.96%
DL2	53,158,430	46,435,548	6,965,332,200	98.56%	96.41%	47.98%
DL1	54,699,692	48,032,432	7,204,864,800	98.58%	96.45%	48.40%

*Note*: KS1, IN and DL1: the scions, interstocks and rootstocks of ‘Kanshu’/N.x/*Diospyros lotus*; KS2 and DL2: the scions and rootstocks of ‘Kanshu’/*Diospyros lotus*

Functional annotations of unigenes were based on protein sequence similarity, KEGG pathway, COG, and Gene Ontology (GO) analyses. Unigene sequences were queried against protein databases (Nr, SwissProt, KEGG, and COG) using blastx and against the nonredundant NCBI nucleotide (Nt) database using blastn (*E*-value < 0.00001). A total of 91,353 predicted unigenes, which comprised 85,342 (71.37%) unigenes annotated by the Nr database, 60,380 (50.50%) unigenes annotated by SwissProt, 49,032 (41.01%) unigenes annotated by KEGG, 31,219 (26.11%) unigenes annotated by COG, 54,925 unigenes annotated by GO terms (45.93%), and 71,400 (59.71%) unigenes annotated by the Nt database (Table S2). A homology search of the unigenes was performed using the Nr database, which annotated the highest number of unigenes among the six databases. According to the Nr database similarity search, a total of 45.15% of the sequences showed a high degree of similarity (E-values < 1E^−45^), and 54.85% of the sequences showed reasonable similarity (E-values within the range of 1E^−5^ to 1E^−45^); this result indicates a high alignment reliability (Fig. S3a). According to the similarity distribution, 30.87% of the sequences showed more than 80% similarity with sequences in the Nr database, indicating a good function annotation (Fig. S3b). Regarding the sources of matching sequences, *Vitis vinifera* provided the greatest number of blastx matches, matching 24.6% of the total number of unigenes, followed by *Coffea canephora*, *Sesamum indicum*, *Theobroma cacao*, *Indian lotus*, *Citrus sinensis*, and *Nicotiana sylvestris* (Figure S3c).

### GO term analysis

GO analysis provided a functional annotation analysis of all unigenes in *Diospyros kaki*. Figure S4 illustrates the number of unigenes in each GO category. The GO terms for unigenes in the biological processes category included: biological regulation, cellular component organization or biogenesis, cellular, developmental, establishment of localization, growth, localization, metabolic, multicellular organismal, regulation of biological process, response to stimulus, signaling, and single-organism. In the cellular components category, unigenes were classified into cell, cell part, macromolecular complex, membrane, membrane part, organelle, and organelle part. In the molecular function category, *Diospyros kaki* unigenes were classified into binding, catalytic activity, enzyme regulator activity, nucleic acid binding transcription factor activity, structural molecule activity, and transporter activity. According to GO analysis, unigenes were assigned to the biological process terms of biological regulation, metabolic, developmental, signaling and growth, and the molecular function terms of catalytic and enzyme regulator activity after grafting ‘Nantong-xiaofangshi’ as an interstock.

### KEGG analysis

KEGG pathway analysis revealed that 49,032 unigenes in *Diospyros kaki* were mapped to 128 predicted pathways. In the top 20 pathways with the largest number of genes, we observed that most of the unigenes were concentrated in the following pathways: metabolic pathways (ko01100), biosynthesis of secondary metabolites (ko01110), plant hormone signal transduction (ko04075), RNA transport (ko03013), phenylpropanoid biosynthesis (ko00940), and pyruvate metabolism (ko00620). Further analysis of the pathways revealed that the unigenes were also involved in peroxisome (ko04146), tryptophan metabolism (ko00380), diterpenoid biosynthesis (ko00904), zeatin biosynthesis (ko00908), and brassinosteroid biosynthesis (ko00905) pathways (Supplemental data file 1).

### Phytohormone levels and water conductance

**Fig. 3:**
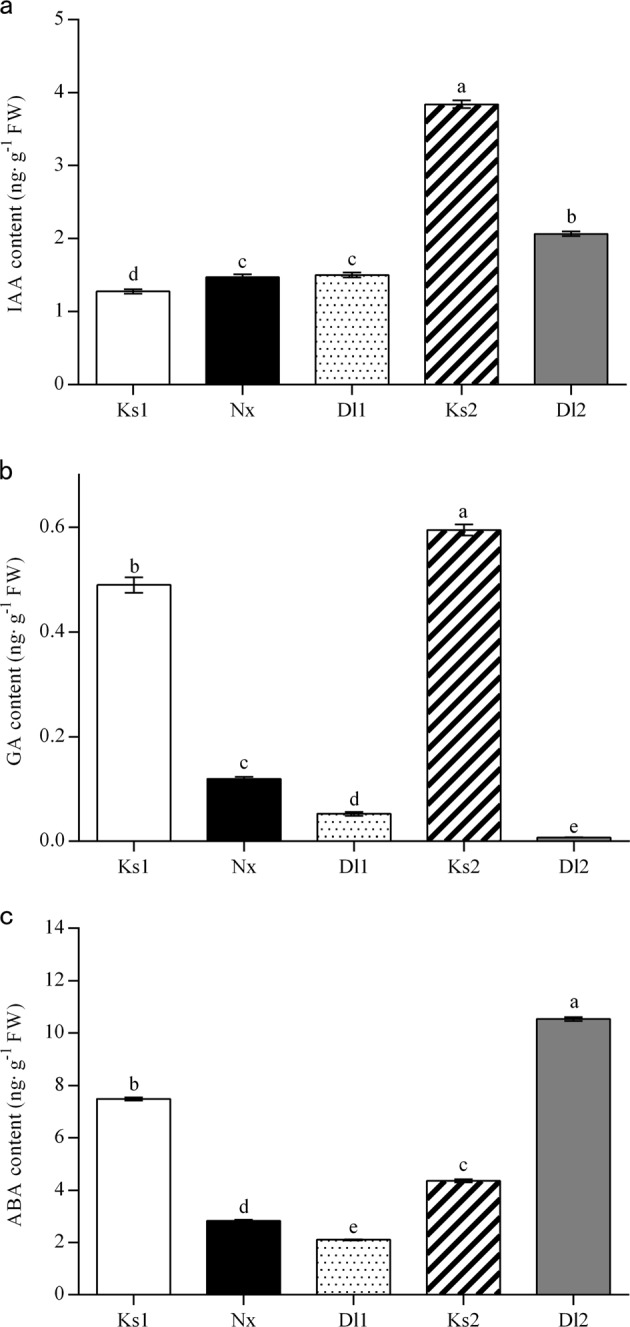
Phytohormone levels in different tissues of ‘Kanshu’ persimmon with different graft combinations. **a** Indole-3-acetic acid (IAA), (**b**) gibberellin (GA_4_), and (**c**) abscisic acid (ABA). Ks1, Nx, and Dl1 represent ‘Kanshu’, ‘Nantong-xiaofangshi’, and *Diospyros lotus*, respectively, in ‘Kanshu’/‘Nantong-xiaofangshi’/*Diospyros lotus* grafting plants. Ks2 and Dl2 represent ‘Kanshu’ and *Diospyros lotus*, respectively, in ‘Kanshu’/*Diospyros lotus* grafting plants. Error bars: the standard error of three biological replicates. Different letters: significantly different at *P* < 0.01 by Duncan’s multiple range tests

‘Nantong-xiaofangshi’ interstocks affected hormone levels in the grafted ‘Kanshu’ scions (Fig. 3). IAA levels in scions and rootstocks in the ‘Kanshu’/*Diospyros lotus* graft combination were all significantly higher relative to their levels in the same tissues in the ‘Kanshu’/N.x/*Diospyros lotus* graft combination. In the ‘Kanshu’/N.x/*Diospyros lotus* trees, the IAA content was significantly higher in the interstock tissues than in the scion tissues. In contrast, no difference was observed between the interstock and rootstock tissues (Fig. 3a). GA levels in the scions of trees with ‘Nantong-xiaofangshi’ as an interstock were markedly lower than those in the scions of trees without an interstock. GA levels within the same grafting combination were highest in scion tissues and lowest in rootstock tissues, regardless of whether an interstock was present. This was especially apparent in ‘Kanshu’/N.x/*Diospyros lotus*, where the GA levels in interstock tissues were significantly lower than those in scion tissues but higher than those in rootstock tissues (Fig. 3b). For ABA levels, the results indicated that ABA levels were high in ‘Kanshu’/N.x/*Diospyros lotus* tissues and relatively low in ‘Kanshu’/*Diospyros lotus* tissues (Fig. 3c). In addition, the water conductance of the grafting area was significantly lower in ‘Kanshu’/‘Nantong-xiaofangshi’/*Diospyros lotus* than in ‘Kanshu’/*Diospyros lotus* (Fig. S5).

### RT-qPCR analysis of genes in ‘Kanshu’ scions

**Fig. 4:**
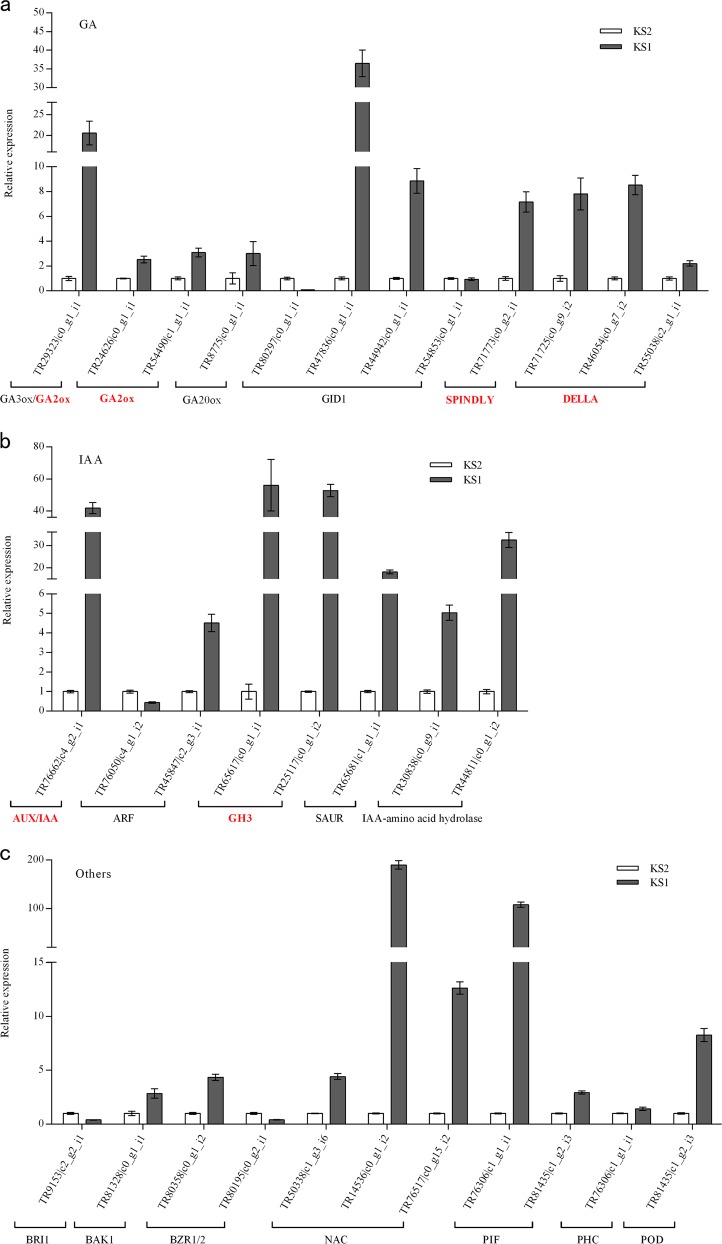
RT-qPCR confirmation of 31 differentially expressed unigenes. **a** Unigenes related to gibberellin (GA) metabolism and signal transduction. **b** Unigenes related to auxin (IAA) signaling. **c** Unigenes related to brassinosteroid (BR), NAC transcription factor, phytochrome-interacting factor (PIF), phenol-containing compound (PHC) and peroxidase (POD). Error bars indicate the standard error of three biological replicates. Note: double asterisk (**) values are significantly different (*P* < 0.01)

According to GO and KEGG analysis, the detected unigenes were related to metabolism pathways, including diterpenoid biosynthesis, tryptophan metabolism, and brassinosteroid biosynthesis pathways, and plant hormone signal transduction pathways, including gibberellin, auxin, and brassinosteroid pathways. Therefore, we analyzed candidate unigenes from these pathways in the dwarfing process with ‘Nantong-xiaofangshi’ as the interstock. These candidate unigenes are very likely associated with the regulation of scion growth by the interstock, and their expression levels were confirmed by RT-qPCR. The results indicated that 31 genes had significant expression differences in ‘Kanshu’ between ‘Kanshu’/*Diospyros lotus* and ‘Kanshu’/‘Nantong-xiaofangshi’/*Diospyros lotus* (Fig. 4).

Regarding GA metabolism, *gibberellin 2beta-dioxygenase* (*GA2ox*) genes (TR24626|c0_g1_i1, TR54490|c0_g1_i1) exhibited higher levels of expression in KS1 than in KS2. Additionally, gibberellin *3beta-dioxygenase* (*GA3ox*) (TR29323|c0_g1_i1) was upregulated in KS1, as was *gibberellin 20-oxidase* (*GA20ox*) (TR8775|c0_g1_i1). Regarding GA signal transduction, *gibberellin insensitive dwarf 1* (*GID1*) gene expression was both upregulated (TR47836|c0_g1_i1, TR44942|c0_g1_i1) and downregulated (TR80297|c0_g1_i1, TR54853|c0_g1_i1) in KS1. Notably, DELLA (DELLA-family proteins) genes exhibited elevated expression in the ‘Kanshu’ scion tissues from the ‘Nantong-xiaofangshi’ graft combination. In contrast to the RNA-seq analysis, the *spindly* gene, which enhances the inhibitory effect of DELLA and negatively regulates gibberellic acid-mediated signaling, was highly upregulated in the KS1 samples relative to the KS2 samples (Fig. 4a). Regarding auxin signal transduction, the expression of *auxin-responsive protein IAA* (AUX/IAA) and *auxin-responsive GH3* gene family members (TR76662|c4_g2_i1; TR65617|c0_g1_i1, TR25117|c0_g1_i2) was elevated in the scion samples from the ‘Nantong-xiaofangshi’ graft combination. Auxin response factor (*ARF*) genes were upregulated (TR45847|c2_g3_i1) in the scion and downregulated (TR76050|c4_g1_i2) in the interstock. The expression of the SAUR family protein (*SAUR*) gene (TR65681|c1_g1_i1) was higher in KS1 than in KS2. Additionally, the transcript levels of two IAA-amino acid hydrolase genes (TR30838|c0_g9_i1, TR44811|c0_g1_i2) were higher in KS1 than in KS2 (Fig. 4b).

### Genetic transformation and functional analysis of *DkGA2ox1* in tobacco

**Fig. 5:**
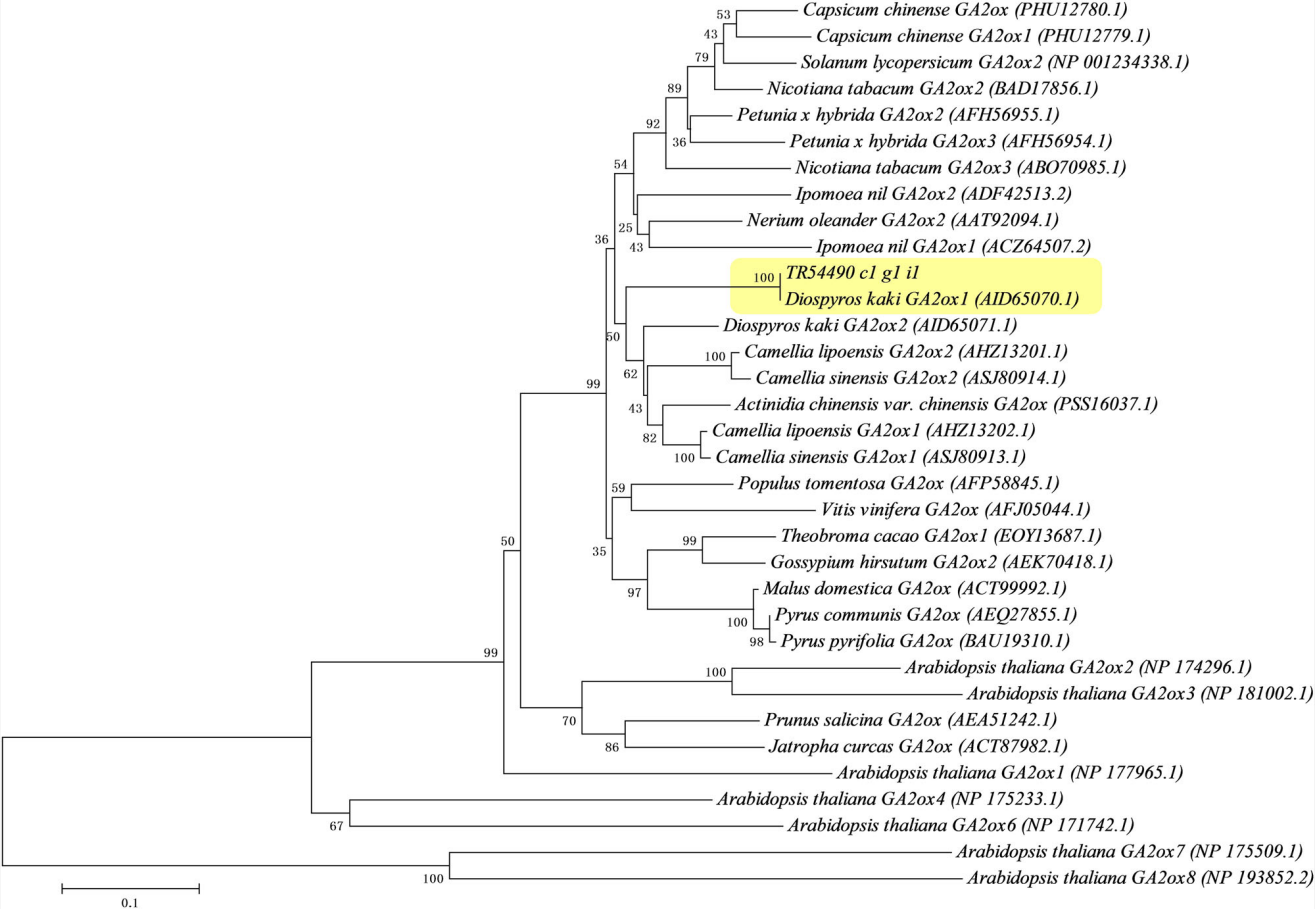
Phylogenetic analysis of *DkGA2ox1*. A neighbor joining phylogenetic tree of TR54490|c1_g1_i1 (*DkGA2ox1*) produced with 1000 bootstrap replications. *DkGA2ox1* is highlighted by a pale yellow box

*DkGA2ox1* (TR54490|c1_g1_i1) expression was elevated in scions (‘Kanshu’) grafted on the ‘Nantong-xiaofangshi’ interstock. The functional role of the expression, however, is unclear. Phylogenetic analysis was used to study the biological function of *DkGA2ox1*. Our phylogenetic analysis suggests that TR54490|c1_g1_i1 (*DkGA2ox1*) is homologous to *Camellia lipoensis GA2ox2* (AHZ13201.1) and *Camellia sinensis GA2ox2* (ASJ80914.1) and belongs to the same subfamily as *Arabidopsis thaliana GA2ox1* (NP177965.1), *GA2ox2* (NP174296.1) and *GA2ox3* (NP181002.1) (Fig. 5). GA2ox is the principle enzyme involved in the inactivation of bioactive GAs, and GA plays a critical role in stem growth and elongation. Therefore, a functional analysis of this gene was conducted in transgenic tobacco. The results indicated that *DkGA2ox1* was successfully integrated into the tobacco genome and was expressed in the transgenic lines of tobacco (Fig. S6). Two independent transgenic tobacco lines were selected for further study.

**Fig. 6:**
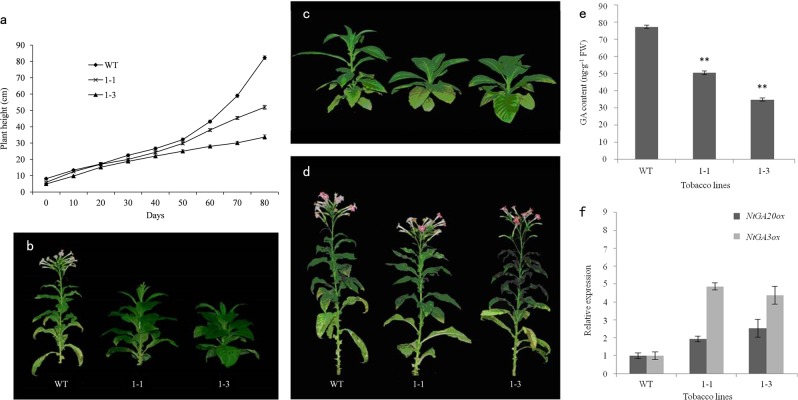
Genetic transformation and functional analysis of DkGA2ox1 in tobacco. **a** Plant height of tobacco. **b** Tobacco plants 90 days after planting. **c** Transgenic plants before GA_3_ treatment. **d** Reversal of normal phenotype of transgenic plants by GA_3_ treatment 30 days after transplanting. **e** GA content in transgenic and wild-type tobacco. **f** Expression of associated genes in transgenic and wild-type tobacco. WT: nontransgenic tobacco plant; 1–1,1–3: two independent transgenic lines of *DkGA2ox1*. Note: means with double asterisk (**) are significantly different (*P* < 0.01) by Duncan’s multiple range tests. Each expression analysis utilized three biological and technical replicates. Error bars indicate the standard error of three biological replicates

Compared with the growth of the wild type, the growth of the transgenic lines was slow over 60–90 days after transplanting (Fig. 6a). In addition, the transgenic lines exhibited significant dwarfism, compact leaf types, and delayed flowering after 90 days of growth in the greenhouse (Fig. 6b). The phenotypic analysis demonstrated that the 1–1 and 1–3 transgenic lines of tobacco were 38.90% and 59.03% shorter than the wild-type plants, respectively. In addition, the internodal length of the transgenic lines was 13.15–60.30% shorter than the internode length of the wild-type plants. In contrast, the stem diameter in the transgenic lines was 11.92–57.42% greater than that in the wild-type plants. Finally, leaf length was 23.27–47.07% shorter in the transgenic lines, while leaf width increased by 21.79–25.00% (Table 3).

**Table 3:** Phenotype characteristics of *DkGA2ox1* transgenic tobacco

	WT	*DkGA2ox1*	
		1-1	1-3
Plant height (cm)	82.33 ± 2.59	50.3 ± 2.69**	33.73 ± 1.33**
Internodal length (cm)	4.03 ± 0.12	3.5 ± 0.1 **	1.6 ± 0.1**
Stem diameter (mm)	9.23 ± 0.15	10.33 ± 0.15**	14.53 ± 0.47**
Leaf length (cm)	24.37 ± 1.59	18.70 ± 0.55**	12.9 ± 0.66**
Leaf width (cm)	8.4 ± 0.26	10.5 ± 0.1**	10.23 ± 0.15**
Leaf length/width	2.9 ± 0.10	1.78 ± 0.04**	1.26 ± 0.03**

*Note*: means with double asterisk (**) are significantly different (*P* < 0.01)

WT: nontransgenic tobacco plant; 1–1,1–3: transgenic tobacco plants of *DkGA2ox1*

The transgenic and wild-type tobacco plants received two applications of 100 mg·l^−1^ GA_3_ each week starting at 30 days after transplantation to the greenhouse. Thirty days after the first application of exogenous GA_3_, the internode length in the transgenic plants began to increase rapidly, and treated plants flowered normally (Fig. 6c, d). Thus, it appeared that the GA_3_ application restored the normal phenotype to the transgenic plants.

GA levels were significantly lower in the transgenic lines than in wild-type plants. Overall, the transgenic GA levels were 65–70% of the levels observed in wild-type plants (Fig. 6e). We suggest that the overexpression of *DkGA2ox1* in tobacco effectively inhibited the synthesis of GA in the plant, which resulted in the production of shorter plants. Additionally, we analyzed the gene expression of several key enzymes that are involved in GA biosynthesis. The heterologous overexpression of *DkGA2ox1* enhanced the expression of the native *NtGA20ox* and *NtGA3ox* genes in transgenic tobacco plants compared to wild-type plants, and the increased expression of *NtGA3ox* was more evident than that of *NtGA20ox* (Fig. 6f).

### Cleaved amplified polymorphic sequence analysis of the *GA2ox* gene

**Fig. 7:**
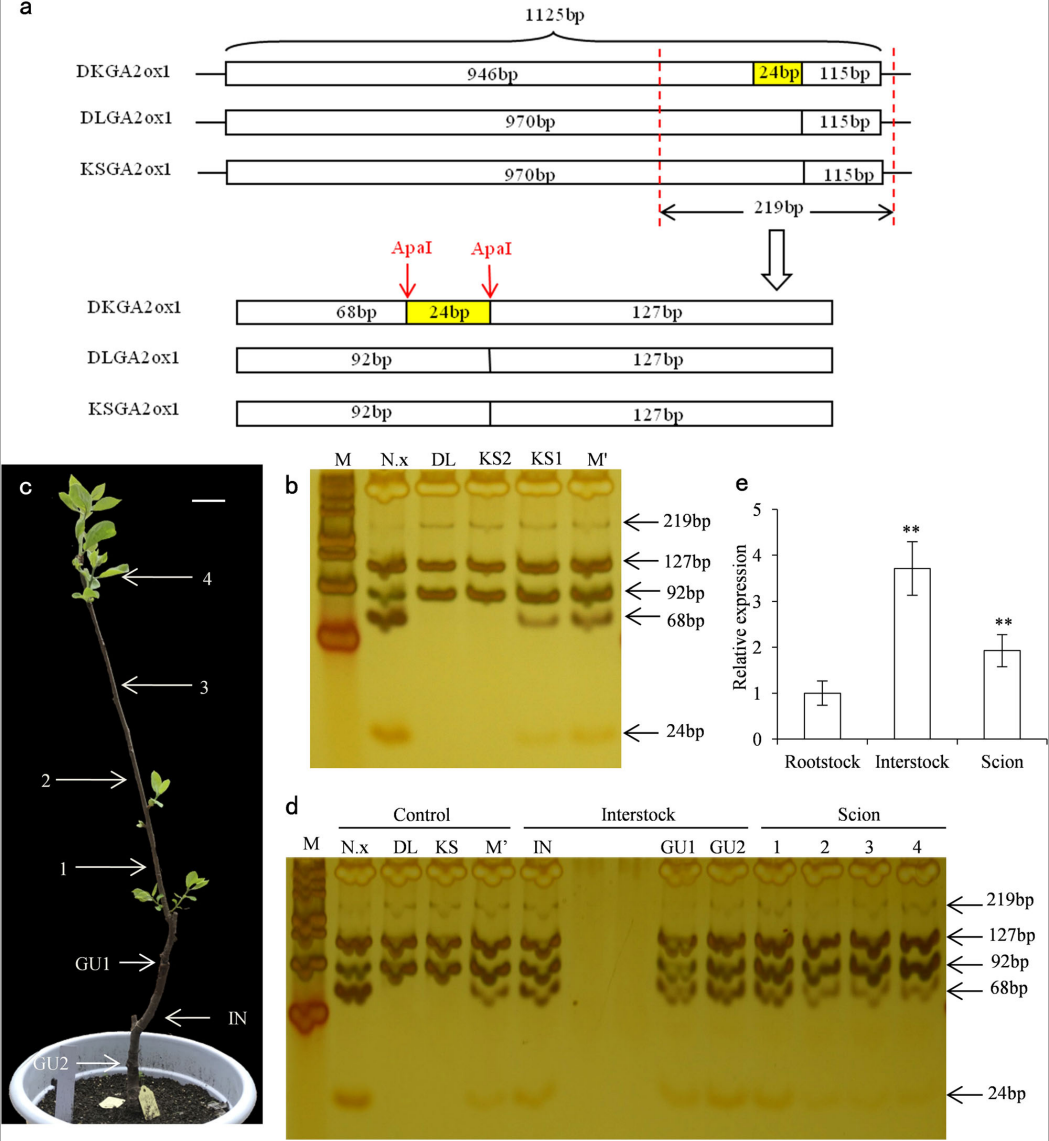
Cleaved amplified polymorphic sequence (CAPS) analysis of RT-PCR products of the persimmons. **a** Fragment sizes predicted by ApaI digestion in ‘Nantong-xiaofangshi’, *Diospyros lotus* and ‘Kanshu’. **b** Polyacrylamide gel electrophoresis of restrictive digestion of different RT-PCR products. **c** Samples from an 82 cm tall grafted tree with ‘Kanshu’ as scion, ‘Nantong-xiaofangshi’ as interstock and *Diospyros lotus* as rootstock. **d** CAPS analysis on purified RT-PCR products of different tissues in ‘Kanshu’/‘Nantong-xiaofangshi’/*Diospyros lotus*. **e** Comparison of total *DkGA2ox1*/*DLGA2ox1*/*KSGA2ox1* transcripts in ‘Kanshu’/‘Nantong-xiaofangshi’/*Diospyros lotus*. *DkGA2ox1*, *DLGA2ox1* and *KSGA2ox1* are the sequences in ‘Nantong-xiaofangshi’, *Diospyros lotus* and ‘Kanshu’, respectively; N.x: ‘Nantong-xiaofangshi’/*Diospyros lotus*, DL: *Diospyros lotus*, KS: ‘Kanshu’, M’: mixture of cDNA from N.x, DL and KS2, KS1: ‘Kanshu’/‘Nantong-xiaofangshi’/*Diospyros lotus*, KS2: ‘Kanshu’/*Diospyros lotus*, GU1: graft union of *Diospyros lotus* and ‘Nantong-xiaofangshi’, IN: ‘Nantong-xiaofangshi’ interstock, GU2: graft union of ‘Nantong-xiaofangshi’ and ‘Kanshu’, 1-3: ‘Kanshu’ scion every 10 cm from the GU2, 4: young leaves at the top of the scion. M: DNA ladder marker 500. *Scale bars* = 5 cm. Note: double asterisk (**) indicates a significant difference (*P* < 0.01). The purified PCR products were diluted to the same concentration for enzyme cutting

*GA2ox1* genes were amplified from *Diospyros lotus*, ‘Nantong-xiaofangshi’, and ‘Kanshu’ tissue samples based on the sequence of *DkGA2ox1*. The polypeptides encoded by these genes comprise 332 amino acid residues, including regions composed of PLN02156 (gibberellin 2-beta-dioxygenase), Pcbc, (Isopenicillin N synthase and related dioxygenases), DIOXˍN (non-haem dioxygenase in morphine synthesis N-terminal), and 2OG-FeIIˍOxy (2OG-Fe(II) oxygenase superfamily). The genes shared 99.26% sequence identity and contained 25 single nucleotide polymorphisms (Fig. S7). As minor differences were difficult to distinguish by RT-qPCR, restriction enzyme sites within the sequences were identified, and the ApaI cut site was used to distinguish among *DkGA2ox1* (‘Nantong-xiaofangshi’), *DLGA2ox1* (*Diospyros lotus*), and *KSGA2ox1* (‘Kanshu’). The enzyme digest fragments are shown in Fig. 7a.

Since the fragment used to distinguish the genes was only 24 bp, a cleaved amplified polymorphism sequence (CAPS) marker was designed to distinguish *DkGA2ox1* from the other two sequences. The CAPS marker allowed us to demonstrate that the *DkGA2ox1* transcript was transported from the interstock (‘Nantong-xiaofangshi’) to the scion (‘Kanshu’). The 219 bp fragment amplified from N.x cDNA was cleaved into three fragments of 24, 68, and 127 bp, while the fragment amplified from ‘Kanshu’ and *Diospyros lotus* cDNA was cleaved into two fragments of 92 and 127 bp (Fig. 7a). RT-PCR restriction fragment length polymorphism (RFLP) analysis of ‘Nantong-xiaofangshi’ revealed a unique restriction band pattern when subjected to polyacrylamide gel electrophoresis (Fig. 7b lanes 2-4). The 92 bp fragment in ‘Nantong-xiaofangshi’ may occur due to incomplete enzyme digestion or may contain both mRNAs of GA2ox. Relative to trees without an interstock (KS2) (Fig. 7b lane 4), CAPS analysis exhibited 24, 68, 92, 127, and 219 bp fragments in the scion of the ‘Kanshu’/N.x/*Diospyros lotus* graft combination (KS1) (Fig. 7b lane 5). This indicated that the scion had incorporated fragments originating from both the scion and the interstock plants. *DkGA2ox1* transcripts produced in the interstock can be transported from the N.x interstock to the ‘Kanshu’ scion.

To further examine the transmission of *DkG2ox1* transcripts within grafted plants, samples from a twice grafted, 82 cm tall ‘Kanshu’/N.x/*Diospyros lotus* tree were collected from the scion every 10 cm. In addition, samples were collected from the two graft unions and interstocks (Fig. 7c). *DkGA2ox1* transcripts were PCR amplified and purified for CAPS analysis to discriminate SNPs in different locations. Fragments of 24 bp and 68 bp, which were present in the ‘Nantong-xiaofangshi’ interstock, were detected in the two graft unions, young leaves at the top of the scion, and scion tissues at different distances from the graft union. These results indicate that endogenous *DkGA2ox1* mRNA originating in the interstock can be transported from the interstock to 10–40 cm across the graft union into the scion tissues (Fig. 7d). Moreover, in the graft combination ‘Kanshu’/‘Nantong-xiaofangshi’/*Diospyros lotus*, the total expression of *DkGA2ox1*, *DLGA2ox1*, and *KSGA2ox1* (TR54490|c1_g1_i1) was highest in the interstock tissue samples (Fig. 7e).

## Discussion

The growth and development of plants are regulated in a coordinated manner by phytohormones and environmental factors^62^. Plant hormones such as IAA, GA, ABA, CTK, and BR participate in regulating plant growth. Many studies have reported that plant dwarfing is a result of the differential expression or interruption of genes that play a role in phytohormone metabolism^63^ and signal transduction^64^. Despite the use of ‘Nantong-xiaofangshi’ as an interstock in sweet persimmon dwarfing cultivation, the physiological and molecular change caused by this interstock is poorly understood. Moreover, some studies have shown that xylem vessel characteristics appear to determine the hydraulic conductance capacity and influence vegetative growth^7^. Morphological analysis of the graft junction showed the effective sealing of the ‘Kanshu’ scion with the interstock and rootstock (Fig. S8). The phloem and most of the xylem were completely sealed at the graft junctions, which had a low effect on transport between the scion and interstock/rootstock. Interestingly, the water conductance in the tree with ‘Nantong-xiaofangshi’ as an interstock was markedly lower than that in trees without an interstock (Fig. S5), and this decreased conductance could reduce substance transport and influence phytohormone levels in the scion. In addition, the hormone contents in scions grafted to the interstock could reflect the water conductance under less stressful conditions (Fig. 3). This result indicated that the number of graft unions and the interstock variety probably also affected the water conductance of the grafting area to a certain extent, and the low water conductance in the grafts with interstocks could be a major reason for dwarfing in response to ‘Nantong-xiaofangshi’ interstock grafting.

The effect of grafting on plants was evident throughout the entire year, and over the course of multiple seasons, the plant populations were stable. The seasonal change in the growth speed of plants differed, and the most obvious change was observed during the rapid elongation of shoots. At the end of April and during early May, the scion growth showed obvious differences between the two grafted combinations. This period is one of the stages in which the interstock has a significant effect on growth. In the present study, GO and KEGG analyses of unigenes revealed that the unigenes were involved in pathways including plant hormone signal transduction and metabolism. Unigenes in the pathways of diterpenoid biosynthesis, tryptophan metabolism, and GA, IAA hormone signal transduction are probably the candidate genes causing the dwarfing effect of interstocks on tree growth. Plant hormones and the expression of hormone-related genes significantly changed in scion tissues when ‘Nantong-xiaofangshi’ was used as an interstock (Figs. 3, 4). This result indicates that the ‘Nantong-xiaofangshi” interstock plays an important role in modifying the balance of phytohormones in grafted plants and also indicates that the physical distance to the shoot meristem might affect transcript and hormone levels.

Even if the physical distance to the shoot meristem has an impact on plants, the IAA and GA levels in KS1 (grafted with an interstock) were still lower than those in KS2 (grafted without an interstock). Therefore, this point may not be the main reason for the change in hormones in the tissue for research. In the present study, we primarily focused on the role of IAA and GA, as well as their related genes, in the dwarfing effect of the ‘Nantong-xiaofangshi’ interstock.

IAA has a profound influence on the regulation of scion growth by an interstock or rootstock^2,13^. In this study, IAA levels in the scion and rootstock were lower in plants where ‘Nantong-xiaofangshi’ was used as an interstock than in those with no interstock (Fig. 3a). Michalczuk^64^ also detected a low IAA level in scions after they were grafted on a dwarf rootstock^65^. Early isotope labeling experiments demonstrated that dwarfing rootstocks block IAA transport to roots and that the content of IAA received by the roots of dwarfing rootstocks is lower than that in roots of vigorous rootstocks^20^. The polar transport of IAA can be impeded by dwarfing interstocks and result in the production of a dwarf phenotype^13^. The dwarfing mechanism of interstocks vs. rootstocks may be different, but both can reduce the level of IAA present in scions^66^. We hypothesize that the ‘Nantong-xiaofangshi’ interstock influenced the distribution of IAA and its transport to the rootstock, thereby resulting in the dwarfing of the scion.

Auxin can induce the expression of several auxin-responsive genes that fall into three different categories: *Aux/IAA, GH3*, and *SAUR* gene families, as well as ARF genes, the principle target of auxin^67^. The expression of *AUX/IAA* and *GH3* genes was increasingly downregulated with increasing IAA levels and vigorous growth (Fig. 4b). This downregulation was due to the enhanced expression of *AUX/IAA* genes, which act as repressors of auxin transduction and GH3 proteins, deactivating IAA and impeding auxin signaling by catalyzing the disassociation of IAA^68^. Liu et al.^12^ investigated gene expression in ‘Shatangju’ mandarin grafted on different rootstocks and demonstrated that the expression of *GH3* genes and the *AUX/IAA* family gene, *IAA4*, was downregulated as growth and vigor increased^8^. Based on the abovementioned studies and the role of auxin in the regulation of plant growth, the altered expression of *AUX/IAA* and *GH3* family genes probably plays a crucial role in dwarfing. Notably, several *ARF* genes were upregulated and downregulated in our study. One possible interpretation of the contrasting expression profiles in response to the same signal is that *ARF* genes may play diverse functions in plants. Five of the *ARF*s (*ARF5-8 and 19*) in *Arabidopsis* function as transcriptional activators, while the remaining 18 *ARF*s act as repressors^69^. *ARF* transcription factors may modulate *GH3* genes, whose expression is correlated with plant growth and development^70^. This premise is in agreement with the results of our qPCR analysis (Fig. 4b). Upregulated expression of *ARF* genes may stimulate *GH3* transcription in plants using ‘Nantong-xiaofangshi’ as an interstock, thereby hindering the transmission of auxin signaling and leading to dwarfing of the scion. The expression patterns of *ARF* genes need further investigation.

GA promotes stem elongation, and its effect is closely related to IAA, i.e., IAA acts with GA in a synergistic manner, and GA affects the activity of IAA^71^. In the current study, GA levels in the scion and interstock tissues of ‘Kanshu’/‘Nantong-xiaofangshi’/*Diospyros lotus* grafted plants were lower than those in the scion tissues of ‘Kanshu’/*Diospyros lotus* grafted plants. A negative correlation was observed between the GA levels and scion dwarfing (Fig. 3b). GAs can be transported over long distances in grafted plants and can regulate growth^22,23^. As previously mentioned, dwarfing rootstocks and interstocks can decrease the basipetal transport of IAA to the roots. Hooijdonk et al.^23,24^ confirmed this premise and further indicated that GA synthesis and transmission are also suppressed by rootstocks, resulting in decreased levels of GA in the scion and in dwarfed plants. We found a positive correlation between the levels of GA and IAA in the scion tissues of the two graft combinations. Auxin promotes the synthesis and transduction of GA, which is necessary for the biosynthesis of active forms of GA^71^. Exogenous application of an auxin transport inhibitor has been shown to remarkably decrease the GA levels in shoots^72^. Therefore, we hypothesize that GA synthesis and transport to aboveground plant parts (scions) are limited by the ‘Nantong-xiaofangshi’ interstock, which thus reduces GA levels in the scion and results in a dwarf phenotype.

In addition to hormone analysis, we examined gene expression in different tissues to further understand the dwarfing mechanism. GA2ox can catalyze the conversion of bioactive GA and its precursors into irreversible forms of inactive GA^73^. Liu et al.^8^ reported that the expression of *GA2ox1* was negatively correlated with plant growth and vigor. *GA2ox* may act as a substitute for *GID1* in the deactivation of bioactive gibberellins with a high affinity^47^. In our study, the relatively low levels of GA in the scion of ‘Kanshu’/‘Nantong-xiaofangshi’/*Diospyros lotus* grafted plants were probably caused by the upregulation of *GA2ox* in the scion tissues, which may have led to the inactivation of bioactive GA (Fig. 3b). *GA20ox* and *GA3ox* regulate the last stage of GA biosynthesis and thus control the level of bioactive GA^74^. Interestingly, the expression of both *GA20ox* and *GA3ox* increased in the scions of plants where ‘Nantong-xiaofangshi’ was used as an interstock (Fig. 4a). Ou et al.^74^ also reported that *GA3ox* expression levels were not downregulated in dwarf varieties^75^. These results indicate that *GA20ox* and *GA3ox* expression are not the only factors impacting the level of GA. The flux of active GA is due to the balance between the rate of bioactive GA synthesis and deactivation. Therefore, if the GA inactivation rate is faster than the GA synthesis rate, low levels of GA are likely to be observed in the scion of ‘Kanshu’/‘Nantong-xiaofangshi’/*Diospyros lotus* grafted plants.

Notable GA signal transduction-related unigenes were those encoding DELLA proteins, which act as central repressors of GA signaling^74^. GA signaling can be moderated by the gibberellic acid insensitive (GAI) repressor, which possesses the highly conserved DELLA domain. *Atgai* overexpressing *Arabidopsis* plants exhibit a dwarf phenotype^42^. After extensive research, Li and colleagues demonstrated that *GAI* mRNA can be transported in phloem tissues across a graft union and induce a dwarf phenotype in grafted scions^36,37,42^. Therefore, we speculate that a ‘Kanshu’ scion grafted on a ‘Nantong-xiaofangshi’ interstock accumulates more DELLA transcripts that without an interstock, and this inhibits the transmission of GA signals. This process may be partially responsible for the dwarfing of the scion in persimmon when ‘Nantong-xiaofangshi’ is used as an interstock. Our analysis of gene expression also indicated that the expression of a gene encoding *SPINDLY* (*SPY*) was similar to the expression of *DELLA* genes (Fig. 4a). *SPINDLY* (*SPY*) also plays an important role in repressing GA-induced stem elongation^76^. The negative regulation of the GA response pathway by the *SPY* gene is accomplished by the activation of the suppressive function of DELLA proteins^76^. Therefore, the expression of *SPY* may activate DELLA proteins, thereby affecting GA signaling and inducing the dwarf phenotype.

*GA2ox* is currently considered to promote the deactivation of bioactive GAs, or their precursors, to inactive forms. *GA2ox* genes have been studied in several species, including *Arabidopsis*^77^, rice^78^, *Populus*^79^, and plum^47^, in which *GA2ox* expression strongly impeded the biosynthesis of bioactive GA and was associated with a dwarf phenotype. Previous research has identified several mechanisms for GA inactivation, the most prevalent of which is 2β-hydroxylation. GA2oxs, which are responsible for this activity, are divided into two groups: one acts on C19-GAs, and the other acts on C20-GAs^21^. GA2oxs acting on C19-GAs to form inactive products can be further divided into two subgroups according to substrate^80^. The first subgroup deactivates 3β-hydroxy bioactive compounds (GA_1_ and GA_4_), including *AtGA2ox1-3*, and the other group acts on C19-GA immediate non3β-hydroxylated precursors (GA_9_ and GA_20_), including *AtGA2ox4,6*^77^. The GA2oxs acting on C20-GAs (GA_12_ and GA_53_) are considered as a separate family from C19-GA2oxs and include *AtGA2ox7-8*^77^. Based on sequence phylogeny, *DkGA2ox1* belongs to the first subgroup, with C19-GAs as substrates (Fig. 5).

In the present study, GUS detection and RT-PCR analyses indicated that the exogenous *DkGA2ox1* gene was transferred into tobacco successfully and was not present in the wild-type plant. The expression of *DkGA2ox1* in transgenic tobacco resulted in a dwarf phenotype and other dwarfing traits that could be overcome by the exogenous application of GA_3_ (Fig. 6a-e). These results confirmed that the dwarf phenotype was caused by the overexpression of *DkGA2ox1*, which reduced bioactive GA levels. The overexpression of *AtGA2ox7* and *AtGA2ox8* in *Arabidopsis* and *OsGA2ox6* and *OsGA2ox5-OX* in rice resulted in similar dwarfing characteristics^77,78^. Additionally, expression analysis of GA-synthesis genes indicated that *GA20ox* and *GA3ox* are upregulated in GA-deficient and GA-insensitive mutants^81,82^. In our study of transgenic tobacco, the expression of native *NtGA20ox* and *NtGA3ox* genes was upregulated in transgenic lines overexpressing *DkGA2ox1* (Fig. 6f). This was probably due to a feedback regulation mechanism induced by the decrease in the level of endogenous GA.

Some mobile macromolecules, such as RNAs and proteins, have been shown to be transported in the vascular system and pass through a graft union to their targets in the grafted scion^31,83,84^. To further explore the role of *GA2ox* in the dwarfing mechanism of the ‘Nantong-xiaofangshi’ interstock, we determined whether *GA2ox* mRNA was transported from the interstock to the scion, as that may be one of the mechanisms responsible for the dwarfing of the scion. Studies have shown that many different mRNAs can be transported across the graft union in adult plants^5,34,85^. Therefore, we used adult grafted persimmon plants as our experimental material. The results indicated that native *DkGA2ox1* mRNA was transported from the ‘Nantong-xiaofangshi’ interstock to the ‘Kanshu’ scion (Fig. 7). El-Sharkawy et al.^47^ grafted ‘Early Golden’ (EG) on ‘DGO24’, a dwarf plum hybrid rootstock, and observed high levels of *PslGA2ox* expression, which enhanced the accumulation of *PslGA2ox* in the scion and resulted in a compact stature phenotype in the scions of grafted trees. They suggested that the ‘DGO24’ rootstock may be involved in the inactivation of bioactive GAs within the scion, a premise that was supported by the low amount of active GAs in EG/D trees relative to control trees^47^. Increased expression of *GA2ox1* was observed in the scion grafted onto the ‘Nantong-xiaofangshi’ interstock (Fig. 4a). This indicates that the ‘Nantong-xiaofangshi’ interstock can enhance the accumulation *GA2ox1* in the scion, and the high expression of *GA2ox1* in KS1 may be affected by the transport of *DkGA2ox1* transcripts.

In the ‘Kanshu’/‘Nantong-xiaofangshi’/*Diospyros lotus* grafted plants, the content of GA in the interstock was much lower than that in the scion tissues. This may be due to the significantly higher expression of *GA2ox1* in the interstock than in the scion tissues (Figs. 3b, 7e). In addition, the GA level in the rootstock with interstock was higher than that in the rootstock without interstock (Fig. 4a). These observations suggest that GA transported from the rootstock may be deactivated in the interstock, thus affecting the bioactive GA content in the scion. GA levels in the scion were higher than those in the interstock, but this may be due to the nondirectional transport of GA, with equal movement of GA in both an acropetal and a basipetal direction^86^. Furthermore, *DkGA2ox1* mRNA produced in the interstock can be transported to scion stems and leaves at least 10–30 cm away from the graft union in twice-grafted trees (Fig. 7c, d). This suggests that the transport of *DkGA2ox1* mRNA may be similar to the transport of *GAI* and *NAC* mRNA, which was also shown to be transported long distances from the source to sink tissues where they were accumulated^36,64,87^. The transport of mRNA in vascular tissues is a complex biological process. Although there are differences between herbaceous and woody plants, future experiments using the generated *DkGA2ox1*-overexpressing transgenic tobacco plants and wild-type tobacco plants to test whether grafting causes dwarfing of wild-type tobacco scion can further clarify this process.

In conclusion, the ‘Nantong-xiaofangshi’ interstock governed the metabolism, transport, and distribution of endogenous hormones, resulting in the reduced growth (dwarfing) of the grafted scion. In grafted plants, water conductance mostly impacted dwarfing by the interstock because of substance transport, including the transport of phytohormones to the scion, primarily depends on the xylem stream. Additionally, the phytohormone signaling level in the scion could reflect the dwarfing phenotype, which is possibly affected by water conductance. The dwarfing interstock altered gene expression associated with the metabolism and signal transduction of IAA and GA and influenced differential phytohormone levels in the two different graft combinations. Regarding the differential expression of the hormone-related genes, we observed that the *GA2ox* gene was highly expressed in the scion. One of the *GA2ox* genes was annotated as *DkGA2ox1* in *Diospyros kaki*, and the overexpression of this gene in transgenic plants resulted in a dwarf phenotype. Furthermore, *DkGA2ox1* transcripts originating in the interstock plant part were identified in the scion, thus indicating that the transport of *DkGA2ox1* transcripts could have an additional effect on dwarfing. However, the contribution of mobile transcripts is still unknown. These pathways may cooperatively cause the dwarfing phenomenon exhibited when a dwarfing interstock is utilized. Our studies lay the foundation for additional studies on the dwarfing mechanism of the ‘Nantong-xiaofangshi’ interstock, in which the details of the proposed mechanisms of dwarfing are fully elucidated and demonstrated.

## Supplementary information

The supplementary Tables and Figures

KEGG analysis of all unigenes in *Diospyros kaki*
