# Supplementary material for: Transcriptomic analysis of interstock-induced dwarfism in Sweet Persimmon (Diospyros kaki Thunb.)
Source: Hortic Res. 2019 May 1;6:51. doi: 10.1038/s41438-019-0133-7 (PMC6491603; doi:10.1038/s41438-019-0133-7)
Supplement: Supplementary file 1 — The supplementary Tables and Figures [file 41438_2019_133_MOESM1_ESM.docx]

**Supporting information**

**Transcriptomic Ana****lysis of Interstock-Induced Dwarfism in Sweet** **Persimmon (*Diospyros Kaki* Thunb.)**

Yanying Shen^1^, Weibing Zhuang^2^, Xutong Tu^1^, Xinyi Yu^1^, Xuehan Li^1^, Feihong Li^1^, Shenchun Qu^1*^

^*^ Corresponding authors

^1^ College of Horticulture, Nanjing Agricultural University, Nanjing, Jiangsu, 210095, China

^2^ Institute of Botany, Jiangsu Province and Chinese Academy of Sciences, Nanjing, Jiangsu, 210014, China

| **Table S1****. Gene specific primer pairs for qRT-PCR.** | | |
| --- | --- | --- |
| Gene ID | Putative name and function | Forward/ Reverse primer sequences (5'-3') |
| TR29323\|c0_g1_i1 | gibberellin 3-oxidase/gibberellin 2-oxidase | 5'-AAGATGGGTCACTGGTCG-3' |
|  |  | 5'-GGAAGTCGCTGTCGGTAT-3' |
| TR24626\|c0_g1_i1 | gibberellin 2-oxidase | 5'-GCACAACTCCTTCTTCATC-3' |
|  |  | 5'-CTTCTCCTCTCCTTCTTCC-3' |
| TR54490\|c1_g1_i1 | gibberellin 2-oxidase | 5'-TCTATCCCACCCGACGAAAG-3' |
|  |  | 5'-AGTGGGGAAAACCATCACGAG-3' |
| TR8775\|c0_g1_i1 | gibberellin 20-oxidase | 5'-CCAACATACCTCCACAGT-3' |
|  |  | 5'-TTGACCACCAGGAAGAAG-3' |
| TR80297\|c0_g1_i1 | gibberellin receptor GID1 | 5'-CGAGAACACCACTTACGA-3' |
|  |  | 5'-CGAGCGGAAGCATCATAA-3' |
| TR47836\|c0_g1_i1 | gibberellin receptor GID1 | 5'-TACCACCTACCTGGATTTCTGTTGC-3' |
|  |  | 5'-ACCACCTTCAGCCTTCTTCA-3' |
| TR44942\|c0_g1_i1 | gibberellin receptor GID1 | 5'-CGGTGATAGGAATGACAAG-3' |
|  |  | 5'-CAGAGGAGAGCAGAGAAT-3' |
| TR54853\|c0_g1_i1 | gibberellin receptor GID1 | 5'-CGTGAGAATGGGAAGAATG-3' |
|  |  | 5'-GGAGATGAAGTGTTGGATAG-3' |
| TR71773\|c0_g2_i1 | polypeptide N-acetylglucosaminyltransferase, SPINDLY | 5'-AGGAACCACCACTACTTG-3' |
|  |  | 5'-GATGTCTGATGCCAACTG-3' |
| TR71725\|c0_g9_i2 | DELLA protein | 5'-ACGGTAGTGGTGAGTAATC-3' |
|  |  | 5'-CAAACAGTGACGAGAAGTG-3' |
| TR46054\|c0_g7_i2 | DELLA protein | 5'-CCAGATGAGAGTGTTACGA-3' |
|  |  | 5'-CGGTGTTGGTATTGGATTC-3' |
| TR55038\|c2_g1_i1 | DELLA protein | 5'-CTGAGAGCCATAGCCATAG-3' |
|  |  | 5'-CAGTGAAGTAAGCAGTAAGC-3' |
| TR76306\|c1_g1_i1 | phytochrome-interacting factor 3 | 5'-AGCCACAGATAGCCATAG-3' |
|  |  | 5'-CTCATCCAGAACCAATGC-3' |
| TR81435\|c1_g2_i3 | phytochrome-interacting factor 3 | 5'-AGAGGGTGAGAAGGGAGA-3' |
|  |  | 5'-GAAGGGATTGGACATAGTG-3' |
| TR76662\|c4_g2_i1 | auxin-responsive protein IAA | 5'-AAAGAAGGCGACTGGATG-3' |
|  |  | 5'-GAAGGGAACAAAGCAACC-3' |
| TR76050\|c4_g1_i2 | auxin response factor | 5'-ATCGCAGCAGAAAGTGAA-3' |
|  |  | 5'-CAGAGGAGGGAAGCAGTC-3' |
| TR45847\|c2_g3_i1 | auxin response factor | 5'-ATGGCACAGACAAGCGATAC-3' |
|  |  | 5'-AATAACTGCTGCTGCGACACG-3' |
| TR65617\|c0_g1_i1 | auxin responsive GH3 gene family | 5'-GCTTGGCTGCTATTATGAG-3' |
|  |  | 5'-GTTGGTCTTGTCGTTGTC-3' |
| TR25117\|c0_g1_i2 | auxin responsive GH3 gene family | 5'-TCCTCGGTCTCTTCTTCT-3' |
|  |  | 5'-CTTGCCTGTTCTGATGTC-3' |
| TR65681\|c1_g1_i1 | SAUR family protein | 5'-TCGTGCCCTACGGCTTAT-3' |
|  |  | 5'-TCCACCACCTTCCAACCA-3' |
| TR30838\|c0_g9_i1 | Iaa-amino acid hydrolase | 5'-CAAGAGCAAGAACGATGG-3' |
|  |  | 5'-GTTGGAAGACGAGTGTGA-3' |
| TR44811\|c0_g1_i2 | Iaa-amino acid hydrolase | 5'-CCACTGGAGCAGCAATAC-3' |
|  |  | 5'-TGCCCTGTGAATCTAACC-3' |
| TR9153\|c2_g2_i1 | protein brassinosteroid insensitive 1 | 5'-CGGAATCTGCTTGAGTTG-3' |
|  |  | 5'-AAGGAGAGGTTGCCAATC-3' |
| TR81328\|c0_g1_i1 | brassinosteroid insensitive 1-associated receptor kinase 1 | 5'-GCATTCAATCAGCCAACTC-3' |
|  |  | 5'-CCACCACCATCATCATCA-3' |
| TR80358\|c0_g1_i2 | brassinosteroid resistant 1/2 | 5'-GGCTGATGTTCCAATGTC-3' |
|  |  | 5'-TTCTGCTTCCTCTTCCTG-3' |
| TR80195\|c0_g2_i1 | brassinosteroid resistant 1/2 | 5'-ATCACAGGACGCAGCAAG-3' |
|  |  | 5'-ACCATCTCCGCATAAACC-3' |
| TR50338\|c1_g3_i6 | NAC transcription factor | 5'-GAAAGACTGATTGGGTGATG-3' |
|  |  | 5'-ATGGAGGAAGAGATGAAGAG-3' |
| TR14536\|c0_g1_i2 | NAC domain-containing protein | 5'-GGCGAGTCTAATCCTGTG-3' |
|  |  | 5'-CGAAAGTTGGTATCCGTGT-3' |
| TR76517\|c0_g15_i2 | NAC domain-containing protein | 5'-TTCTTCTCCATTCTACCAGG-3' |
|  |  | 5'-CATATCAACACCGTCCATTC-3' |
| TR29743\|c0_g1_i1 | ribulose bisphosphate carboxylase/oxygenase activase | 5'-ACCTTGTATGCTCCTCTG-3' |
|  |  | 5'-TCCACTTCCTCACTTCATC-3' |
| TR70982\|c0_g1_i1 | peroxidase | 5'-CAATGCCGATGACTGAAC-3' |
|  |  | 5'-TGAGAGAAGCCGATAGTG-3' |

**Table S2. Unigenes annotated in various databases.**

| Database | Number of annotated unigenes | Percentage (%) |
| --- | --- | --- |
| Nr | 85,342 | 71.37 |
| Swiss-Prot | 60,380 | 50.50 |
| KEGG | 49,032 | 41.01 |
| COG | 31,219 | 26.11 |
| GO | 54,925 | 45.93 |
| Nt  All | 71,400  91,353 | 59.71  76.40 |

Note: Nr, non-redundant NCBI protein database; Swiss-Prot, Swiss-Prot protein database; KEGG, Kyoto Encyclopedia of Genes and Genomes; COG, Clusters of Orthologous Groups; GO, Gene Ontology; Nt, non-redundant NCBI nucleotide database.


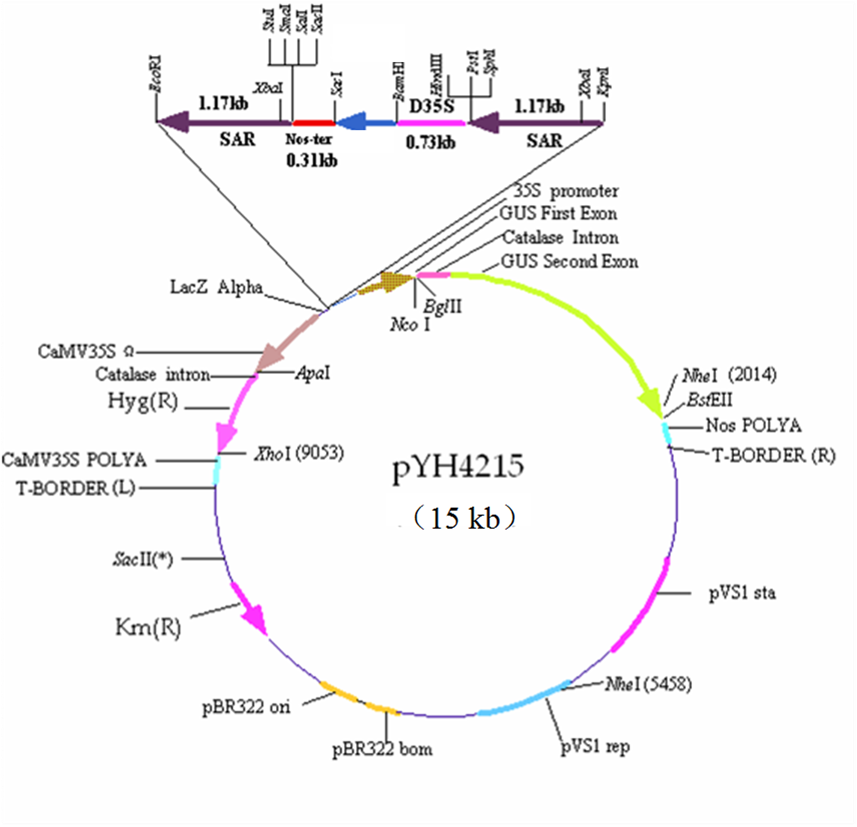


**Figure S1. Construct of plant overexpression vectors of pYH4215.**

**
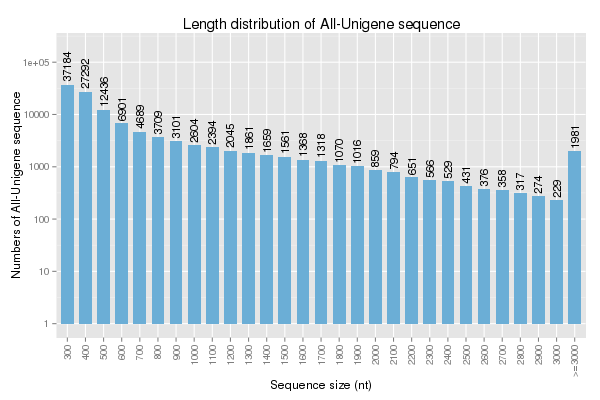
**

**Figure S2. Length distribution of unigenes in *Diospyros kaki* transcriptome.**


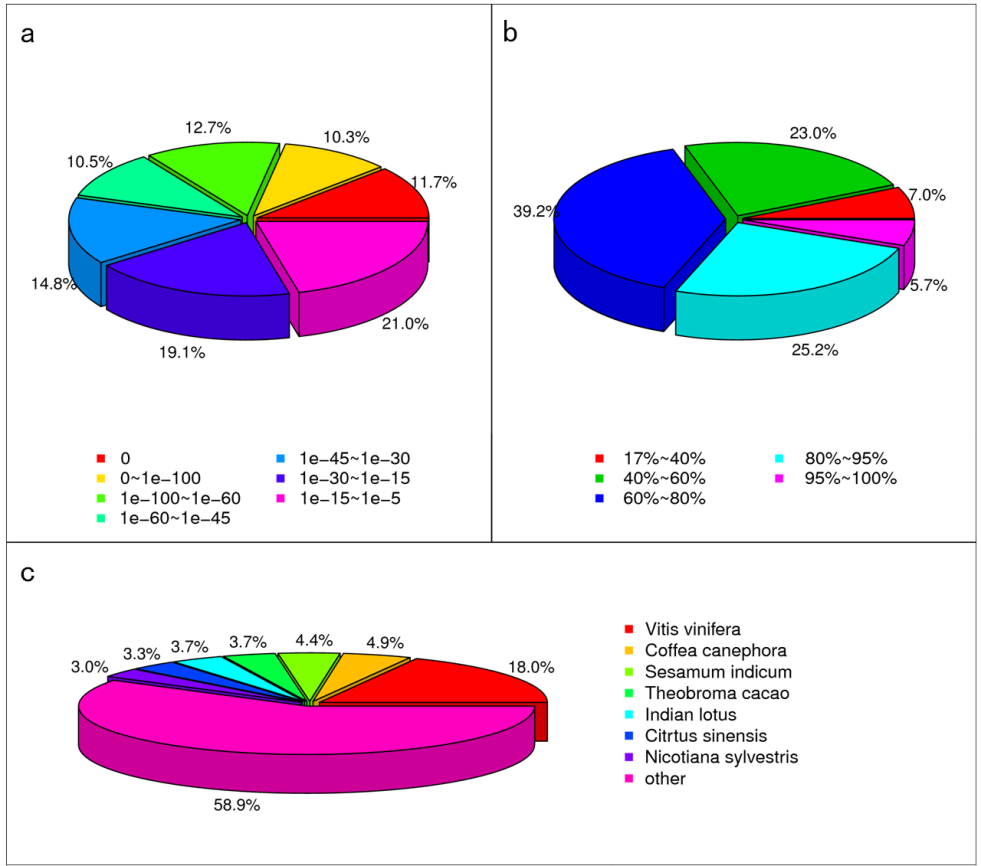


**Figure S3. Unigene annotation results according to the NR database. (a) The E-value distribution. (b)** **The similarity distribution. (c) The species distribution (E-value < 1.0e-5).**


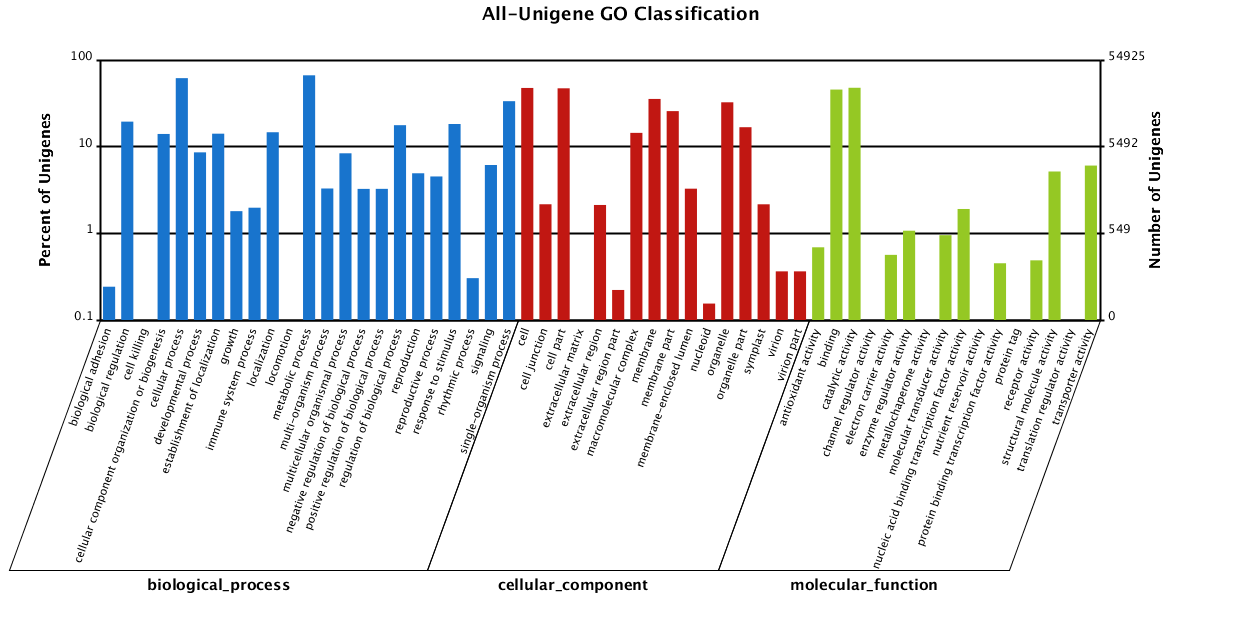


**Figure S4. GO classification of unigenes in *Diospyros kaki*. The horizontal axis: types of GO function; the right vertical axis: number of unigenes annotated to the GO function; the left vertical axis: unigenes as percentage of the total.**

**
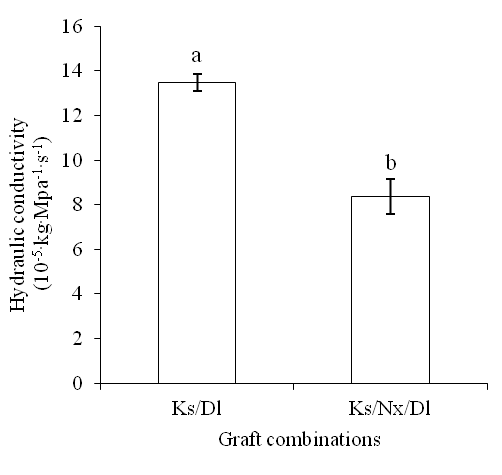
**

**Figure S5.** **Hydraulic** **conductance of grafting area for persimmon trees on two graft combinations. Ks/Dl:** **'Kanshu'/*Diospyros lotus*, Ks/Nx/Dl:** **'Kanshu'/'Nantong-xiaofangshi'/*Diospyros lotus*, Different letters: significant difference tests were used to compare individual treatment means (*P* < 0.05).** **Error bars: the standard error of three biological replicates.**


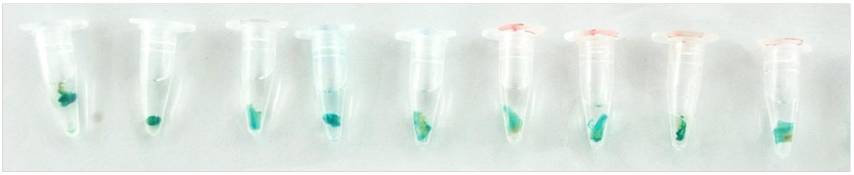


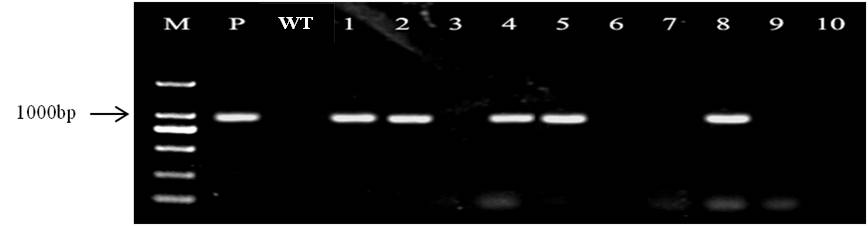


**Figure S6. GUS straining of Hyg-resistance tobacco plants and detection of the *DkGA2ox1* gene mRNA transcripts by RT-PCR. M: molecular size marker; P: positive control (plasmid DNA); WT: non-transgenic tobacco plant; 1-10: transgenic tobacco plants.**

**Figure S7. Sequence Alignment of *GA2ox1* gene in** ***Diospyros lotus*** **(*DL-GA2ox1*), 'Nantong-xiaofangshi' (*DKGA2ox1*), and 'Kanshu' (*GS-GA2ox1*).**

**
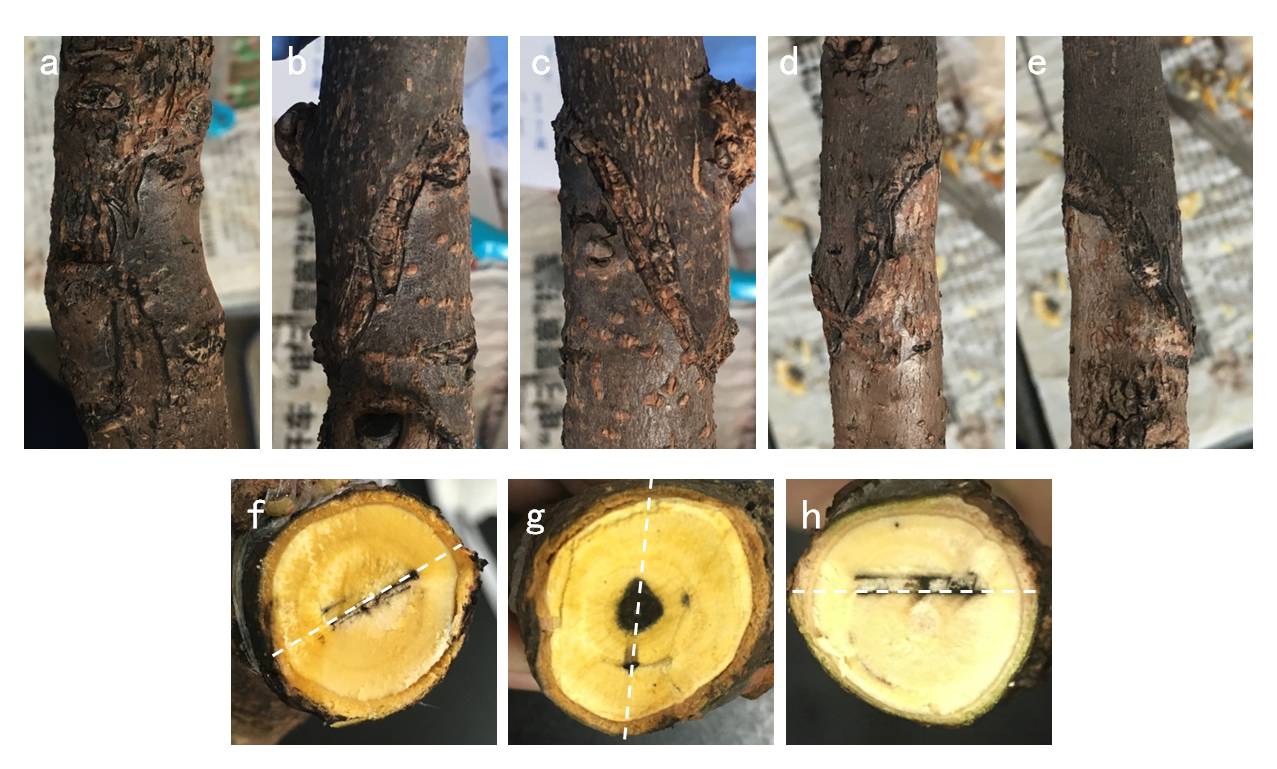
**

**Figure S8.** **The morphological analysis of graft junctions on** **'Kanshu'/'Nantong-xiaofangshi'/*Diospyros lotus* (a-c, f-g) and** **'Kanshu'/*Diospyros lotus* (d-e, h). (a) the profile of graft junction between rootstock and interstock; (b-c) the profile of graft junction between interstock and scion; (d-e) the profile of graft junction between rootstock and scion;** **(f)** **the transection of graft junction between *Diospyros lotus* and 'Nantong-xiaofangshi' in** **'Kanshu'/****'Nantong-xiaofangshi'/*****Diospyros lotus*; (g) the transection of graft junction between 'Nantong-xiaofangshi' and 'Kanshu' in** **'Kanshu'/****'Nantong-xiaofangshi'/*Diospyros lotus*; (h)** **the transection of graft junction between *Diospyros lotus* and 'Kanshu' in** **'Kanshu'/*****Diospyros lotus*.** **The white dotted line was graft union boundary.**

**Supplemental data file 1.xls:** KEGG analysis of all unigenes in *Diospyros kaki*.
